# A Blueprint for Connection: Mapping Interconnected Patterns of Relationship Change in Couples Using the Agapé App †

**DOI:** 10.3390/bs16071182

**Published:** 2026-07-13

**Authors:** Ronald D. Rogge, Jenna A. Macri, Khadesha Okwudili, Dev Crasta

**Affiliations:** 1 Department of Psychology, University of Rochester, 462 Meliora Hall, RC Box 270266, Rochester, NY 14627, USA; jenna.macri001@umb.edu (J.A.M.); dev.crasta@va.gov (D.C.); 2 Agapé Wellness Inc., Rochester, NY 10016, USA; khadesha@theagapeapp.com; 3 Center of Excellence for Suicide Prevention, Department of Veterans Affairs, Canandaigua, NY 14424, USA

**Keywords:** relationship enhancement, relationship wellness, romantic relationships, couples, marriage, relationship dynamics, mechanisms, gratitude, partner responsiveness, quality time

## Abstract

Agapé is a light–touch relationship enhancement smartphone app. This study used data from a longitudinal study of couples using the Agapé app to explore change within an array of behavioral processes to uncover patterns of interconnected change, thereby providing some of the first quantitative insights into how various relationship processes might be linked as relationships change over time. A sample of 405 couples in long-term relationships (810 partners, 50% women, 75% white, together M = 4.5 yrs, 50% living together, 33% currently dissatisfied) completed assessments across their first month of using Agapé. Men and women significantly improved on 15 of the 16 relationship processes assessed. As the study lacked a randomized control condition, it remains unclear if those improvements were due to using the Agapé app or to factors like expectancy effects, regression to the mean, self-selection, demand characteristics, or general participation effects. Network analyses explored correlational linkages among the self-reported pre–post changes observed. Results highlighted increases in three processes (quality time spent together, perceived partner responsiveness, and gratitude toward partner) as the processes most proximally linked to increases in relationship quality. The network findings also uncovered a number of patterns of interconnected change to be explored in future studies (e.g., increases in couples talking about their relationships to increases in mindful awareness within those relationships to increases in gratitude and quality time to increases in relationship quality). Thus, the results offer some of the first comprehensive multivariate (albeit correlational) insights toward understanding how relationship processes might work in concert with one another within a broader multivariate pattern of self-reported relationship change.

## 1. Introduction

Marital discord and divorce are significant social issues that disrupt the lives of families and children (Amato, 2000), costing billions of dollars per year in the United States alone (Schramm, 2006). Despite decades of research developing a host of effective relationship enhancement programs to improve relationship functioning (Rolffs & Rogge, 2016), limited work has examined processes of change in these programs (Markman et al., 2022), creating a critical gap in our understanding. Basic research into relationship functioning has identified several key relationship processes associated with relationship satisfaction and longevity (Karney & Bradbury, 1995) and intervention research suggests that many of these processes can change simultaneously (Le et al., 2020). However, further theory development and refinement of interventions will require a more cohesive understanding of how these different processes may work in concert with one another, potentially organizing themselves into chains or cascades to shape relationship quality.

### 1.1. Approaches to Relationship Enhancement (RE)

A host of effective relationship-strengthening interventions has been developed to prevent discord and divorce (Markman et al., 2022; Rolffs & Rogge, 2016). These range in depth and format from: (1) more intensive 14-h in-person workshops like the Prevention and Relationship Enhancement Program (PREP; Markman et al., 1994), to (2) similar programs with tele-health formats like Couple CARE (Halford et al., 2004), to self-directed online programs like ePREP (Braithwaite & Fincham, 2009, 2011), OurRelationship (Doss et al., 2013, 2016, 2019), and the Promoting Awareness, Improving Relationships program (PAIR; Rogge et al., 2013, 2017). Despite their varied formats, these programs generally take a psycho-educational, skill-based approach to strengthening relationships that focuses on raising awareness and promoting more adaptive functioning across a set of challenging areas and processes known to be important for relationship health and well-being. To varying degrees, these programs have demonstrated benefits at strengthening relationships and preventing relationship distress and dissolution (e.g., Rogge et al., 2013; for reviews see Markman et al., 2022; Rolffs & Rogge, 2016). However, efforts to scale them to a national level have been limited by practical barriers for in-person interventions (e.g., the need to train specialized facilitators/coaches, scheduling issues and a need for childcare for dyadic and group interventions) and non-use attrition within the self-directed programs (Rogge et al., 2017; Rothman et al., 2019).

### 1.2. The Agapé Smartphone App

The Agapé smartphone app is a light–touch self-directed relationship wellness intervention that was designed to target and maximize user engagement (thereby minimizing non-use attrition) through 7 years of intensive persuasive system design driven by user feedback. The core functionality of the Agapé app is sending daily prompts to both partners of a couple (e.g., “If you were going to give your partner an award, what would it be for, and why?”) and then showing them one another’s responses when they both have answered the prompt. The daily prompts were developed to align with the content of other RE programs and the process was designed to promote moments of connection from the investment of just a few minutes each day. Given the benefits of assessment-based interventions like the Relationship Checkup (Fentz & Trillingsgaard, 2017), the Agapé app also offers users 120-item (8–12 min) wellness checks that provide ongoing feedback on individual and relationship outcomes and a range of relationship processes.

The current study draws upon data from the first single-arm treatment study of Agapé which followed 405 romantic dyads through their first month of using the app. It is important to note that, as this study lacked a randomized no-treatment group, analyses within its data cannot directly control for the potential benefits of anticipatory and placebo effects. Thus, any pre–post benefits observed cannot definitively be attributed to use of the Agapé app as they could simply reflect expectancy effects, regression to the mean, self-selection, demand characteristics, or general participation effects. As a result, the first set of analyses published from the current data was focused primarily on feasibility and acceptability, with additional analyses exploring the possible benefits of using Agapé. Those findings suggested high acceptability and engagement (99% of couples used the app, jointly completing an average of 27 daily prompts across 4 weeks), and correlationally linked more frequent use of the Agapé app to greater gains in relationship satisfaction (Rogge et al., 2024). In contrast, the current analyses employ a novel combination of statistical approaches to deepen our understanding of relationship change by exploring possible linkages among change scores on a broad set of relationship processes.

### 1.3. The Relationship Processes Targeted by RE Programs

As a set, empirically developed RE programs focus on a common set of processes that have been linked to relationship functioning and stability (see Karney & Bradbury, 1995, for a comprehensive review). This includes relationship maintenance behaviors (e.g., Acitelli, 2002) like: (1) learning new things about one’s partner, (2) reflecting on one’s own behavior as a partner, (3) talking with one another about your relationship, (4) spending quality time with one another, and (5) saying “I love you.” Previous work has also highlighted key connective relationship processes, including: (1) providing emotional support (e.g., Sullivan et al., 1998), (2) mindful attentive awareness within relationships (Daks et al., 2021), (3) engaging gratitude (e.g., Algoe et al., 2010), and (4) perceptions of a partner’s responsiveness to your feelings and needs (Reis et al., 2004). Previous work has also uncovered a set of detaching relationship processes that have been linked to the erosion of relationship quality and stability over time: (1) lapses in gratitude (e.g., Lambert & Fincham, 2011), (2) negative conflict behavior (e.g., Rogge & Bradbury, 1999), (3) being distracted from or inattentive toward one’s relationship (e.g., Daks et al., 2021), and (4) a perception that one’s partner is insensitive to one’s own feelings and emotional needs (e.g., Crasta et al., 2021). Finally, previous research has highlighted the importance of a number of sexual functioning processes (e.g., Shaw & Rogge, 2016): (1) sexual activity, (2) physical affection, and (3) sexual satisfaction. Thus, a wealth of both basic and applied research on romantic relationships has identified a diverse set of key relationship processes. Most RE programs, including the Agapé app, take a broadband approach, targeting many if not all of these processes in their efforts to strengthen relationships.

### 1.4. Toward a More Nuanced and Integrative Understanding of Relationship Processes

Despite the wealth of basic and applied research supporting RE programs and their targeted processes, far less research has examined the process of change resulting from RE interventions (Markman et al., 2022), identifying a critical gap in our understanding. This echoes a similar gap found in most psychotherapy research (Kazdin, 2009). Thus, it remains largely unclear: (1) how the specific RE interventions impact a wide range of specific relationship processes, (2) how relationship processes might work in concert (or antagonistically) with one another as relationships grow and change over time, or (3) how improvements in certain processes might be linked to corresponding improvements in closely related (i.e., proximal) processes—potentially forming chains or cascades. This critical gap in our understanding of the underlying, nuanced dynamics of change in romantic relationships limits our ability to optimize RE programs to be maximally effective.

To address this gap, the current analyses sought to use multi-wave longitudinal data from the 405 romantic dyads using the Agapé app (during their first months of use) to examine the unique, correlational links among change in specific aspects of relationship and individual functioning within a larger set of 16 relationship processes, 4 dimensions of relationship quality, and 4 dimensions of individual functioning. This approach therefore offers the possibility of providing comprehensive insights (albeit correlational) into the process of change that might occur in relationships.

### 1.5. A Network Analysis Approach to Examining Processes of Change

As this represents one of the first of its kind to model relationship and individual change across 24 different constructs, we wanted to model change across the relationship processes and outcomes simultaneously. Such analyses would allow us to identify which specific constructs might be more proximally (i.e., directly) or distally (i.e., indirectly) related to one another and to specific outcomes, potentially yielding a more parsimonious, multivariate understanding of the dynamics of relationship change and growth. Given the novelty of examining so many competing processes to understand their links to one another and to changes in relationship functioning, we also wanted to run the analyses in an exploratory manner as it was unclear which sequences of indirect pathways might emerge as dominant. To address these goals, we deviated from the more traditional change mechanism analyses that we preregistered (i.e., correlation and regression) and were inspired by emerging efforts to identify patterns of interconnected change among processes using network analyses (Belli et al., 2026).

### 1.6. Duocentric Network Analyses to Develop Couple-Level Insights

Psychological network analyses extend techniques originally developed for modeling social networks to evaluate the unique links between pairs of variables within a larger set of variables (Epskamp & Fried, 2018). The variables being examined are called nodes (represented as circles in network graphs) and their unique pair-wise associations (i.e., the partial correlations between each pair of variables controlling for all other variables in the model) are called edges. Thus, network analysis represents a method of examining patterns of unique association among a set of variables.

Within social network analyses, researchers have also developed the concept of a duocentric networks, an extension of individual networks (“egocentric”) that combines the reports of a common community from two reporters (Coromina et al., 2008), including extensions to romantic couples reporting on their social networks, which are expected to overlap and connect as both partners are describing a common social community (Kennedy et al., 2015). While each individual network captures individuals’ separate perceptions, integrating the networks has the potential to reveal overlaps and connections between individual perceptions and comparisons between these duocentric networks and egocentric networks may reveal unique relationship processes outside of each individual partner’s awareness (Kennedy et al., 2015). This logic can be extended from social networks to psychological networks. Because both partners are describing the same relationship, their reports would be expected to correlate and connect. Thus, information on relationship dynamics can be integrated across partners to create a duocentric psychological network. Extending the logic of duocentric social networks, integrating psychological networks may reveal associations outside of each partner’s awareness (e.g., one partner’s processes affecting the other). Comparing a duocentric network with individual networks may also help place relationship processes within a broader dyadic context.

### 1.7. Related Work

Compelling findings exploring mechanisms of change emerged in the couples’ literature from analyses in one of the seminal randomized controlled trials (RCTs) to contrast two different cognitive–behavioral therapies for relationship distress (Christensen et al., 2004). Multi-level modeling results examining a handful of potential treatment mechanisms in separate analyses suggested that improvements in positive behaviors, negative behaviors, and more specifically in behaviors the individual partners wanted to target were all predictive of corresponding improvements in relationship quality (Doss et al., 2005).

Turning from the couples’ therapy literature to the RE literature, more recently a number of RCTs have uncovered potential mechanisms of change to explain the benefits of these programs. For example, improvements in intimate safety and acceptance were uniquely linked to increases in relationship satisfaction in an RCT of the Marriage Checkup (Hawrilenko et al., 2016). Multivariate results from an RCT of OurRelationship suggested that improvements in target problem severity were uniquely linked to improvements in relationship satisfaction (Roddy et al., 2020a). Increases in quality time spent together were linked to increases in relationship quality in an RCT of the Within Our Reach program—a modified version of PREP (Carlson et al., 2022). Similarly, improvements in gratitude (Barton et al., 2024) and in negative communication, positive communication, and emotional support (Le et al., 2020) were linked to increases in relationship quality (in separate analyses) for couples participating in either OurRelationship or ePREP within an RCT contrasting each of those RE programs with a control group. Importantly, when taken as a set, these studies examined a relatively small number of candidate mechanisms and largely evaluated them in separate models, leaving unanswered questions regarding how multiple relationship processes might operate in concert with one another.

Shifting from applied couples research to basic studies of relationships, a handful of studies have attempted to link processes to relationship satisfaction in short mechanistic chains. For example, cross-sectional analyses linked higher gratitude indirectly to greater relationship satisfaction through their links to higher perceived partner responsiveness in a sample of 825 online respondents (Jin et al., 2024). Similarly, analyses in longitudinal and experimental studies linked higher gratitude indirectly to increased relationship maintenance through their links to higher positive perceptions of a partner (Lambert & Fincham, 2011). Extending this work, cross-sectional analyses linked greater emotional support (termed dyadic coping in that paper) indirectly to higher relationship satisfaction through their links to higher gratitude in a sample of 163 Swiss couples (Roth et al., 2024). Finally, greater mindful attentive awareness of one’s relationship at a baseline assessment was indirectly linked to increases in relationship quality over two months via the links of those two constructs to higher perceived partner responsiveness during a daily diary assessment between the two timepoints (Stanton et al., 2021). Thus, a growing body of recent work has begun to build a foundation for understanding therapeutic and naturally occurring change within relationships. However, as a set, these studies have largely focused on isolated mechanisms or short mechanistic chains involving only a few relationship processes at a time. This leaves a critical gap in our understanding of how the broader constellation of relationship processes targeted in RE programs works together. More specifically, it remains unclear how they might combine into larger cascades of relationship change. Statistical approaches that can model many interrelated processes simultaneously (like psychological network analysis) could therefore offer unique insights into how relationship change unfolds across interconnected systems of behaviors, perceptions, and experiences.

### 1.8. The Current Study

The current study sought to advance understanding of relationship change—thereby addressing a critical conceptual gap—by taking a more comprehensive multivariate approach. This involved using psychological network analysis to simultaneously model change on a wide range of relationship processes that could be linked to overall change in relationship quality—thereby uncovering the strongest unique correlational links amongst change in those processes. The study draws upon recent work using exploratory psychological network analyses to understand the broader multivariate patterns of change in psychopathology symptoms by uncovering patterns of interconnected change in various depressive and anxiety symptoms at different stages of treatment (Belli et al., 2026). Drawing upon multi-wave data from 405 couples during their first month of using Agapé, the current network analyses examined self-reported change across 16 key relationship processes targeted by the Agapé app and other RE interventions. The analyses sought to identify both proximal links among those processes as well as their proximal links to eight outcomes: 4 dimensions of relationship functioning (relationship satisfaction, positive and negative relationship qualities, and dedication) and 4 dimensions of individual functioning (vitality, quality of life, psychological distress, depressive symptoms). Previously published results from the current study uncovered significant increases in individual functioning and relationship quality over the first month of app use (Rogge et al., 2024). Notably, the lack of a randomized no-treatment control group leaves the underlying source of those improvements unclear as any pre–post benefits observed might reflect non-specific effects (e.g., expectancy, regression to the mean, self-selection, demand characteristics) rather than benefits from using the Agapé app. In light of that limitation, the current study sought to characterize the multivariate pattern of self-reported pre–post improvements observed in the 405 couples using the Agapé app without assuming that change could be attributed to their use of the app. It should also be noted that two of the authors developed the Agapé app and are shareholders in Agapé Wellness Inc. As a result, the study was conducted and this manuscript was crafted under a conflict-of-interest plan and made use of preregistered hypotheses along with transparent procedures, materials, and analytic approaches to help minimize potential biases.

We hypothesized that, consistent with improvements in relationship quality observed, the couples in the study might also have experienced improvements in relationship maintenance, connective processes, sexual functioning, and detaching processes (Hypothesis/Aim 1). We further hypothesized that improvements in those 16 key relationship processes would show unique links to corresponding improvements in the 8 outcomes examined (Hypothesis/Aim 2). Although exploratory, the network analyses could also be expected to uncover patterns of interconnected change suggestive of potential indirect pathways to be tested in future studies (Aim 3). Finally, the network analyses were likely to uncover a handful of relationship processes that seemed to function in a more central manner (showing stronger links to other processes and outcomes), providing initial insights into processes that could potentially serve as effective points of intervention to be explored in future studies (Aim 4).

## 2. Materials and Method

### 2.1. Transparency and Openness

All study materials and procedures were evaluated and approved by a university IRB (Rogge et al., 2024). The study materials are available on the osf.io listing for this project (https://osf.io/vfgke/, accessed on 12 July 2026). The osf.io project listing also includes: (1) a pre-registration for this manuscript (the 2nd paper proposed), (2) our SPSS syntax, (3) our HLM 6.0 syntax and outputs, (4) our R syntax, and (5) our corresponding SPSS, HLM, and R datasets (due to stipulations from the IRB, they are only made available upon reasonable request). Within those materials, we report how we determined our sample size, all data exclusions, all manipulations, and all measures in the study.

### 2.2. Procedure

The following section highlights the study procedures relevant to the current study. For detailed procedures, please refer to the first publication, Rogge et al. (2024).

**The Agapé Smartphone App**. The Agapé app is described in detail in Rogge et al. (2024). Briefly, Agapé is a self-directed relationship wellness app centered on daily dyadic prompts (grounded within the 16 relationship processes common to RE programs) and optional wellness checks. The version examined in the current study had been refined through several years of user-centered development (e.g., van Gemert-Pijnen et al., 2011) and was designed to maximize engagement while preserving broad access through a freemium business model. The app includes over 2000 daily prompts developed and tested within a user base that has organically grown to over 3 million users.

**Assessments**. Partners were sent up to 4 emails and/or provided a link within the app (for the baseline survey only) to complete comprehensive (roughly 30 min) baseline and 1-month surveys (hosted on Qualtrics.com) assessing outcomes as well as the 16 relationship processes addressed by the daily prompts. The baseline survey also collected demographic data, screened for eligibility, and obtained informed consent. All 810 partners completed the baseline survey and 350 partners (43%) completed the 1-month follow-up survey roughly 5.3 weeks after enrollment, providing outcome data for 222 of the 405 couples (55%). In addition to those two primary assessments, partners were sent up to 2 emails every seven days inviting them to complete shorter wellness checks within the Agapé app (120 questions taking 8–12 min) that functioned as weekly diaries. The wellness checks were created and subsequently validated using pilot data from the broader Agape user base. The final items were selected primarily by identifying the 2–4 items of each scale or subscale showing the strongest links to the constructs (based on factor loadings in EFAs and corrected item-to-total correlations in reliability analyses). A total of 611 partners (75%) completed 1617 wellness checks in the 6–7 weeks following baseline, providing longitudinal data for 352 (87%) of couples.

**Attrition**. Attrition analyses are reported in greater detail in Rogge et al. (2024). Briefly, partners who completed the 1-month assessment did not differ from those who did not on 7 of the 8 baseline outcomes examined, with the only significant difference reflecting slightly lower depressive symptoms among completers (d = 0.17). Completers were also somewhat more likely to be White, assigned female at birth, older, married, living together, currently in couples counseling, more highly educated, and from households with higher incomes. These effects were generally small to moderate.

### 2.3. Participants

Eligibility criteria, recruitment procedures, and full sample demographics are reported in Rogge et al. (2024). Briefly, 405 couples were recruited between March and June 2022. Participants were adults in romantic relationships whose partners were also willing to install Agapé, pair their accounts, complete a baseline survey, and allow app-use data to be linked to survey responses. The sample was 50% female and 75% White, with 84% of partners in their 20s and 30s, 50% living together, and couples together an average of 4.6 years. The sample included couples across a wide range of relationship stages and relationship quality, including 33% of partners who were dissatisfied at baseline.

### 2.4. Measures

Unless otherwise indicated, these items used the stem, “In the last week, how often have you…”, were rated on a 6-point scale (e.g., *Not at all* to *Completely*; *Never* to *All of the time*), responses were averaged such that higher scores reflected higher levels of the respective construct, and Cronbach’s alphas were calculated in the current data to evaluate internal consistency.

#### 2.4.1. Relationship and Individual Outcomes

The relationship and individual outcomes were assessed with the same measures reported in Rogge et al. (2024): (1) relationship satisfaction using the 8-item Couples Satisfaction Index (CSI; Funk & Rogge, 2007; *α* = 0.957; using a dissatisfaction cutoff of 27.5), (2 and 3) global relationship qualities using 16-item Positive–Negative Relationship Quality scale (PNRQ; Rogge et al., 2017; *α*_pos_ = 0.968; *α*_neg_ = 0.904), (4) dedication using a 4-item subscale of the Commitment Inventory (CI; Owen et al., 2011; *α* = 0.739), (5) vitality using the 6-item Vitality in Life Scale (VLS; Pollard & Rogge, 2022; *α* = 0.966), (6) quality of life using 9 internally consistent items of the Quality-of-Life Inventory (QOLI; Frisch et al., 1992; *α* = 0.863), (7) psychological distress using 6 internally consistent items of the Mood and Anxiety Symptom Questionnaire (MASQ; Watson et al., 1995; *α* = 0.944), and (8) depressive symptoms using the Patient Health Questionnaire (PHQ-9; Kroenke et al., 2001; *α* = 0.944).

#### 2.4.2. Relationship Maintenance Processes

A larger pool of items assessing basic relationship processes (likely to be impacted by Agapé) was piloted in over 20,000 app users. This pool focused on common everyday behaviors distinct from relationship processes like social support, relational awareness, and gratitude. The sets of items used in the current study had straightforward item content and demonstrated high internal consistencies, stable factor structures, and moderate levels of correlation with each other and with the other targeted relationship processes being assessed, all of which suggest that they are appropriate for multivariate analyses.

**Spending Quality Time Together.** Quality time was assessed with five items developed for the study (“prioritized time with your partner,” “set aside time to do something fun with your partner,” “had fun with your partner,” “actively put your relationship at the top of your priority list,” “enjoyed your partner’s company,” and “gotten each other laughing”; *α* = 0.906).

**Reflecting on Own Behavior.** Two items assessed reflection on own behavior (“reflected on your own behavior in your relationship” and “taken time to think about how you could be a better partner in your relationship”; *α* = 0.803).

**Talking about Your Relationship.** Five items assessed this construct (“talk about your relationship with your spouse,” “tell your spouse what you want from the relationship,” “talk about the quality of your relationship; for example, how good it is, how satisfying it is, or how to improve it,” “discuss and try to work out problems between the two of you,” and “reveal very intimate things about yourself or your personal feelings”; *α* = 0.910).

**Learning New Things about Your Partner.** Two items assessed this construct (“I grew to understand my partner better” and “I learned something about my partner’s thoughts and feelings”; *α* = 0.876).

**Saying, “I love you.”** Two items assessed this construct (“did you tell your partner that you love him/her” and “did your partner tell you that they love you”; (*α* = 0.910).

#### 2.4.3. Connective Relationship Processes

**Providing Emotional Support.** Using the stem, “IN THE LAST WEEK, when your partner was feeling upset, stressed or hassled by some problem or difficult situation, how often did you…” six items of the Support in Intimate Relationships Rating Scale (SIRRS; Dehle et al., 2001) assessed providing emotional support and validation (e.g., “take his/her side when discussing his/her situation,” “say you would feel the same way in his/her situation,” “say you thought he/she handled a situation well”) on a 6-point scale (“Never” to “Always”). Responses were averaged such that higher scores reflected higher support provided (*α* = 0.865).

**Mindful Attentive Awareness of Relationship.** The four items of the awareness subscale of the Attentive Awareness In Relationships Scale (AAIRS; Daks et al., 2021) assessed mindfully attending to one’s relationship (*α* = 0.911).

**Gratitude Engagement toward Partner.** Using the stem, “THINKING OF THE LAST WEEK”, the four items of the Gratitude in Relationships Scale (GRS; Rasmussen et al., 2026) assessed feeling and expressing gratitude toward a romantic partner (e.g., “I found myself filled with gratitude for my partner”; *α* = 0.929).

**Perceived Partner Responsiveness.** The 4-item responsiveness subscale of the Partner Responsiveness and Insensitivity scale (PRI; Crasta et al., 2021) assessed the respondents’ perceptions of the responsiveness of their romantic partners (*α* = 0.915).

#### 2.4.4. Detaching Relationship Processes

**Negative Conflict Behavior.** Using the stem, “IN THE LAST WEEK, when discussing a problem with your partner, how often did you…” six items of the negative conflict subscale of the Aversive Interaction Scale (AIS; Rodrigues, 2010) assessed common negative behaviors (e.g., “yell or scream at your partner,” “swear at your partner,” “mock your partner,” “purposefully insult your partner”). Responses were averaged such that higher scores reflected greater negative conflict behavior (*α* = 0.802).

**Distraction/Inattention toward Relationship.** The four items of the AAIRS (Daks et al., 2021) distraction subscale assessed being distracted and inattentive toward one’s relationship (*α* = 0.911).

**Gratitude Lapses toward Partner.** Four items of the GRS (Rasmussen et al., 2026) assessed failing to experience or express gratitude toward a partner (e.g., “I did not show how thankful I am to my partner”) on a 6-point scale (“Never” to “All of the time”). Responses were averaged (*α* = 0.898).

**Perceived Partner Insensitivity.** A 4-item subscale of the PRI (Crasta et al., 2021) assessed the respondents’ perceptions of their partners’ insensitivity; *α* = 0.886.

#### 2.4.5. Sexual Functioning

As 19% of the couples were in long-distance relationships and 31% lived separately in the same metro areas, the sexual functioning questions within the weekly wellness checks were only shown when individuals indicated on a branching question that they had spent time with their partner in-person within the last week (to allow for physical affection and intimacy).

**Frequency of Sexual Activity.** Three items assessed common (e.g., Shaw & Rogge, 2016) sexual activities (stem: “IN THE LAST WEEK, how often did you do this with your partner…” items: “oral sex,” “vaginal and/or anal sex,” and “other sexual activities”) on a 6-point scale (“Did not happen” to “4+ times per day”). Responses were averaged (*α* = 0.812).

**Frequency of Physical Affection.** Seven items assessed non-sexual physical affection (stem: “IN THE LAST WEEK, how often did you do this with your partner…” items: “cuddling,” “holding one another,” “kissing,” “hugging,” “holding hands,” “touching or caressing,” and “showing affection”) on a 6-point scale (“Did not happen” to “4+ times per day”). Responses were averaged such that higher scores reflected greater affection (*α* = 0.961).

**Sexual Satisfaction.** The six items of a subscale from the Quality of Sex Inventory (QSI; Shaw & Rogge, 2016) assessed sexual satisfaction with a primary partner (*α* = 0.977).

### 2.5. Analytic Strategy

Our analyses follow an emerging approach of using network analyses where changes on key processes are themselves represented as nodes (e.g., Belli et al., 2026), explored both at the individual (i.e., egocentric) and duocentric levels (Kennedy et al., 2015). Thus, we conducted within-sex analyses using individual male and female networks. Then to explore dyadic effects we contrasted the patterns of edge weights produced by these models with two “duocentric” networks that treated all couples as mixed-sex dyads, randomly assigning partners of same-sex dyads to different sex groups. This strategy also aligned our analyses with the majority of the sample as 91% of our couples were mixed-sex couples. Data was cleaned and variables were created in SPSS 29. Network analyses were conducted in *R* (version 4.0.3). The *estimateNetwork* function in the *bootnet* package (Epskamp & Fried, 2021) was used to estimate partial (Spearman) correlation networks with the glasso EBIC approach and LASSO regularization. Finally, we generated visualizations of our models by using the *qgraph* package (Epskamp et al., 2021; version 1.9). We used the HLM program (version 6.0) to obtain slope estimates for each of the constructs.

#### 2.5.1. Network Analyses

Our network analyses were conducted following current best practices (Epskamp & Fried, 2018). Spearman’s rank-correlations were utilized to account for the ordinal nature of Likert-response data. As network analyses are at risk of having elevated rates of Type I error (Brunner & Austin, 2009) leading to lower stability of model estimates (Babyak, 2004), regularization with the Least Absolute Shrinkage and Selection Operator (LASSO; Tibshirani, 1996) is recommended to focus the network analyses on stronger and more stable links, leading to more parsimonious solutions. LASSO regularization applies increasing adjustments to models with larger numbers of parameters and sets notably small edge weights to zero, thereby helping to minimize the interpretation of spurious associations. LASSO regularization was utilized with the Extended Bayesian Information Criterion (EBIC; Chen & Chen, 2008) selection method being used to select the most parsimonious network among them, thereby limiting spurious associations (Epskamp & Fried, 2018). The EBIC hyperparameter, γ, was set to a reasonably conservative 0.3 to favor finding a more parsimonious model.

Following guidance for interpretation of psychological networks (Bringmann et al., 2019), we favored measures of node relevance that accounted for magnitude of edge weights (i.e., strength centrality; expected influence) over measures that emphasized number of connections (e.g., betweenness) that are more suitable to social network analysis. Node-specific centrality metrics have also been used in duocentered social network models without modification (Coromina et al., 2008).

#### 2.5.2. Accuracy and Stability of Edge Estimates

To evaluate the power and relative stability of the network results (i.e., edge weights and indices of centrality), we compared the network results to 1000 simulations (from the netSimulator function of the bootnet package) using varying sample sizes to determine how consistent the results were and the level of sensitivity and specificity offered by various sample sizes for detecting edge weights. We also used 1000 nonparametric bootstrapped samples obtained via the *bootnet* (Epskamp & Fried, 2021; version 1.5) package to obtain confidence intervals for the edge weights estimated in our models. Finally, we used 1000 case-dropping bootstrapped samples (obtained with the bootnet package) to investigate the stability of the indices of centrality within our network models.

## 3. Results

### 3.1. Pre–Post Change

**Figure 1:**
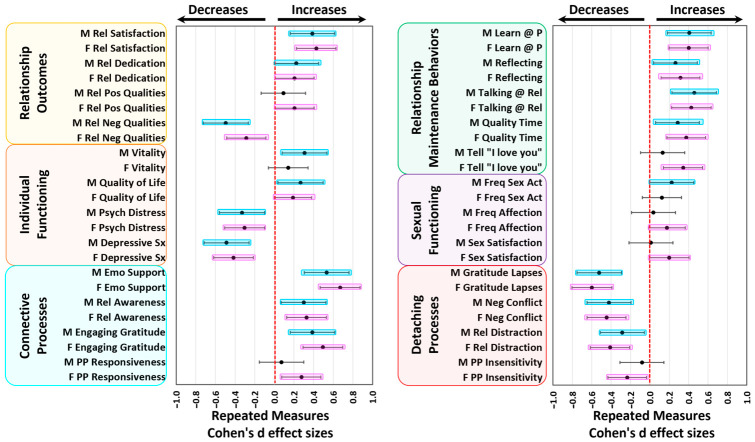
Repeated measures Cohen’s d effect sizes for 1-month pre–post change NOTE: Rel = relationship; Pos = positive; Neg = negative; Psych = psychological; Sx = symptoms; Emo = emotional; and PP = perceived partner. To facilitate interpretation, effects that emerged as significant at *p* < 0.05 from within-person repeated measures ANOVAs (see Supplemental Table S1) are highlighted teal (in men) and pink (in women).

We ran repeated measures Analysis of Variance (rm-ANOVAs) on the 24 constructs examined to look for significant pre–post change. As seen in Figure 1 and the first set of columns of Supplemental Table S1, a series of 2 by 2 rm-ANOVAs treating time (pre vs. post) and partner sex (male vs. female) as within-couple repeated measures identified significant pre–post change on all eight outcomes and on 15 of the 16 processes (failing to uncover significant change on sexual satisfaction). Notably, only two time by sex interactions emerged as marginally significant, suggesting that men and women reported comparable average change across a majority of the constructs examined. Taken together, these results offer partial support for Hypothesis 1, suggesting that the couples reported improvement on the dimensions examined during the first month of using Agapé.

### 3.2. Linking Pre–Post Change

#### 3.2.1. Collinearity and Accuracy/Stability Checks

We ran network analyses on the raw change scores for our 24 constructs, including baseline scores on relationship satisfaction to help control for baseline differences in relationship quality. The network analyses in men and women notably simplified the correlation matrix by uncovering a far smaller set of unique links (called edge weights) between pairs of variables (i.e., partial correlations between pairs of variables, controlling for all other variables in the analysis, Supplemental Figure S5). Simulation analyses suggested that with a sample size of 220 for the pre–post network analyses in men and women, the edge weights generated in the simulated datasets correlated extremely strongly (i.e., average correlations of roughly 0.80 to 0.90) with the estimates in our data (Supplemental Figure S1A,B), as did the estimates of centrality (Supplemental Figure S2A,B). The bootstrapped confidence intervals for the edge weights (Supplemental Figure S3A,B) suggested reasonable precision of the network solutions, and case-dropping bootstrapping analyses further suggested that dropping as much as 30% of the sample still yielded estimates of centrality that, on average, correlated 0.80 or higher with the centrality estimates in the full sample (Supplemental Figure S4A,B). These analyses demonstrated similar support for the dyadic models tested.

#### 3.2.2. Network Visualization—Spring Plots

When plotting network analysis results as spring plots, variables (or nodes) are represented as circles and their unique associations with other variables (i.e., their edges) are represented as lines. The thickness and color depth/intensity of the lines reflect the strength of those unique associations (i.e., the relative edge weights), and the color of the lines reflects the directions of association (i.e., blue—positive associations; red—negative associations). Finally, spring plots use an algorithm to arrange the nodes in order of centrality with more central nodes being placed closer to the center of the diagram.

#### 3.2.3. Results in Individual Spring Plots

**Figure 2:**
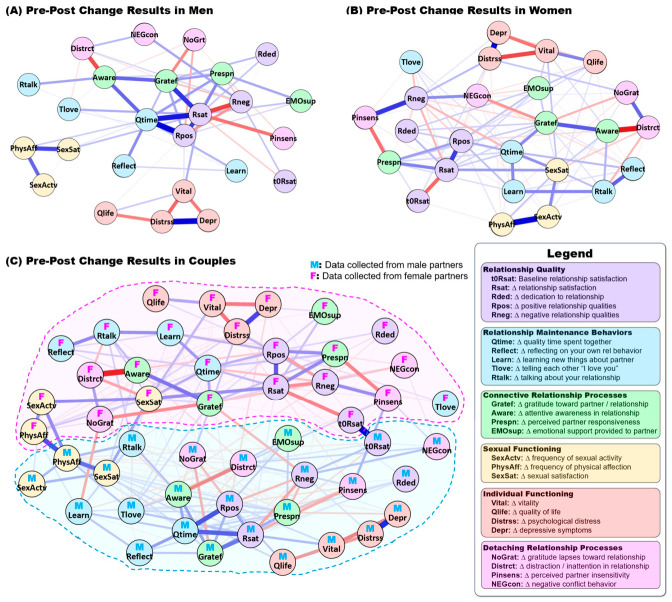
Network analysis results on pre–post change in men (**A**) and women (**B**) separately, and as a dyad (**C**). NOTE: The variables in the models (nodes) are represented by circles. The lines connecting the nodes (edges) represent partial correlations between each pair of variables, controlling for all other variables in the network. The color of the edges indicates the direction of those unique associations (red for negative links, blue for positive links) and the width and color intensity of the edges reflect their relative strengths (thicker lines with more saturated colors reflecting stronger links). The spring plots graph the results empirically by placing nodes with greater centrality (e.g., those with stronger and greater numbers of edges) nearer the center of the graph.

The spring plot in men (Figure 2A) and women (Figure 2B) suggested that pre–post change on the relationship outcomes of satisfaction and positive relationship qualities emerged as some of the most central outcomes in the network. Notably, change on those outcomes demonstrated proximal links to change on quality time spent together, engaging in gratitude toward partner, and perceived partner responsiveness (constructs also emerging as notably central to both networks). This begins to suggest that improvement on those specific processes might occupy intermediate positions between global improvements and improvements in more distal processes assessed in the current model. Tracing some of the strongest unique link edge weights in the model shows similar patterns in men/women, highlighting possible indirect pathways to be explored in future studies. For example, increases in relationship talk and reductions in distraction/inattention from the relationship were both associated with increased mindful attentive awareness, which in turn was linked to feelings of gratitude toward those partners. In contrast, increases in saying, “I love you” to partners demonstrated a simple direct link to increases in relationship satisfaction. Differences also emerged in the patterns of association between men and women’s models. For example, while change in the four individual functioning measures and on the three sexual functioning measures showed strong links within each of the sets of variables, change on those sets of variables only displayed a small handful of unique links to the rest of the model in men while showing more unique ties to change on other relationship processes in women.

#### 3.2.4. Dyadic Results

**Figure 3:**
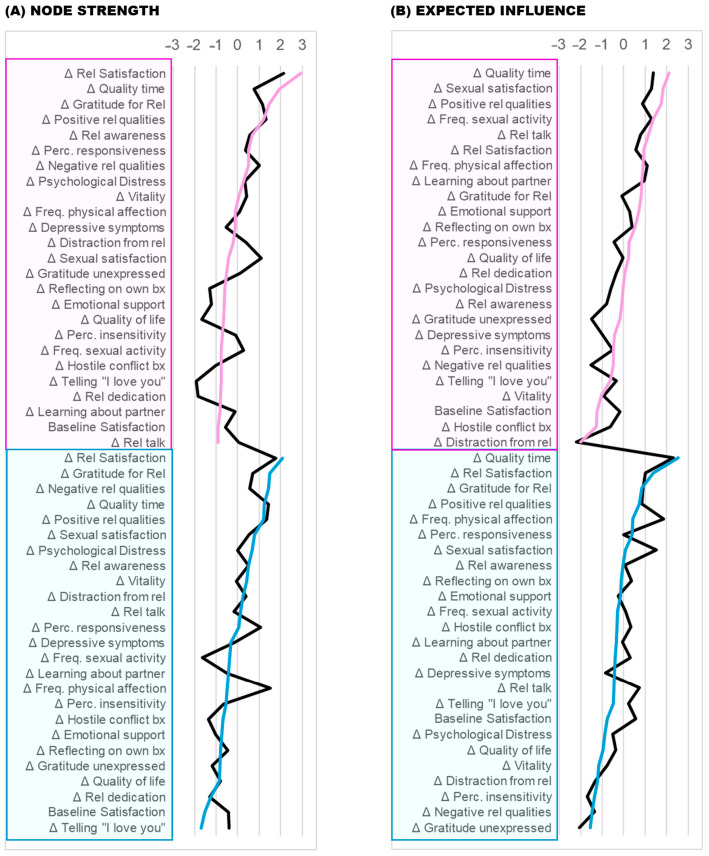
Estimates from the pre–post network models of node Strength (**A**) and expected influence (**B**). NOTE: Strength is the sum of absolute edge weights and therefore reflects the overall magnitude of unique associations with other variables for each construct. Expected influence is the sum of the direct/indirect paths between a given node and all other nodes (multiplying node signs through each of these paths), thereby representing the overall activation or deactivation of the model driven by change in a single node. These are estimated both for individual networks of variables within women (pink lines, corresponding to Figure 2B) and men (blue lines, corresponding to Figure 2A), and in the dyadic networks (black lines, Figure 2C).

Given the dyadic nature of the sample, we ran a third (couple-level) network model examining unique links among change on the constructs in men and women simultaneously (see Supplemental Figure S6 for the full correlation matrix across the change scores and the resulting edge weights from the network analysis). As seen in the spring plot (Figure 2C), both men and women’s patterns of associations were reasonably replicated as two parallel “communities” of variables. However, in these duocentric models, we also found important points of connection between the two spring plots that notably alter interpretation of the centrality in the duocentric model, representing the strong correlations between models. To highlight these differences further and focus on variables that substantially impact relationships to other variables, we overlaid the standardized centrality estimates from the two individual models onto the estimates from the duocentric model (Figure 3). For example, men’s physical affection showed a standard deviation higher of strength centrality and expected influence in the duocentric model than the individual models, highlighting the strong connections between increases in men’s affection and associated increases in women’s sexual activity. Similarly, men telling their partner “I love you,” while rather non-central in their individual model, had higher levels of strength and expected influence in the duocentric model due to many diffuse, low-magnitude links to varied components of women’s relationship functioning (e.g., perceptions of quality time, their own gratitude engagement, and physical affection). Finally, baseline satisfaction for both partners had higher levels of couples’ “starting points” in their relationship before they began their Agapé process. In contrast, individual functioning variables within both men and women retained roughly the same, lower levels of centrality in the duocentric model, reflecting that changes in each partner’s individual functioning were primarily only associated with changes in their own individual experiences of that relationship.

### 3.3. Linking Linear Trajectories of Change

#### 3.3.1. HLM Multi-Wave Slope Strategy

To make full use of the 2777 waves of longitudinal data collected, including the weekly wellness checks, we ran a three-level HLM model. This approach allowed us to retain nearly the full sample. Thus, we ran three-level (repeated assessments nested within individual partners, nested within couples) Hierarchical Linear Modeling (HLM) analyses estimating linear slopes of change across the month of the study (setting linear change as a random effect at level 2—the level of individual partners) on the smaller set of 15 constructs assessed within those wellness checks (3 outcomes and 12 relationship processes). We then ran network analyses on those slope estimates to identify unique links between change processes, building one comprehensive duocentric model examining weekly change in men and women simultaneously.

#### 3.3.2. HLM Multi-Wave Slope Results

**Figure 4:**
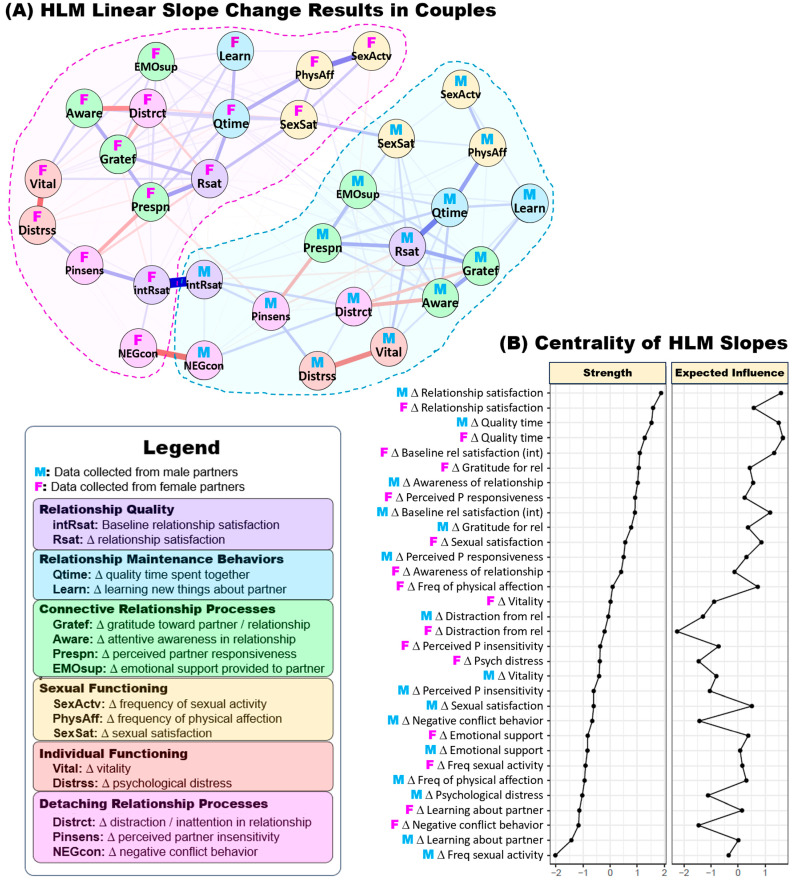
Dyadic network analysis HLM slopes of linear change (**A**) and strength centrality indices (**B**). Notes. The variables in the models (nodes) are represented by circles. The lines connecting the nodes (edges) represent partial correlations between each pair of variables, controlling for all other variables in the network. The color of the edges indicates the direction of those unique associations (red for negative links, blue for positive links) and the width and color intensity of the edges reflect their relative strengths (thicker lines with more saturated colors reflecting stronger links). The spring plots graph the results empirically by placing nodes with greater centrality (e.g., those with stronger links to other nodes and greater numbers of edges) nearer the center of the graph.

As seen in the spring plot (Figure 4) and in the edge weights (Supplemental Figure S7), although a few stronger partner effects (i.e., linking change over time between partners) emerged in the final model, a majority of the retained edges were actor effects (i.e., occurring within men or within women), resulting in male and female networks that were somewhat independent of each other. Linear changes in relationship satisfaction continued to emerge as the central constructs in both men and women, showing robust proximal links to changes in quality time spent together, feeling grateful toward a partner, and perceived partner responsiveness. Although these analyses contained a slightly smaller number of relationship processes, they continued to suggest roughly similar indirect pathways to be explored in future studies. Specifically, they continued to highlight that changes in connective processes were more central to relationship satisfaction within individuals, fluctuations in individual functioning processes were less central to satisfaction, and that sexual functioning and conflict emerged as notable “bridges” between men’s and women’s changing experiences over the course of their relationship.

## 4. Discussion

Decades of research have developed a wide range of effective relationship strengthening interventions (Markman et al., 2022; Rolffs & Rogge, 2016) to help prevent marital discord and divorce. Unfortunately, the impact of those interventions has been limited, in part, by our narrow understanding of the mechanisms underlying relationship change (Markman et al., 2022). The current findings begin to address these challenges by exploring unique linkages between 1-month change scores and trajectories on a set of 16 distinct relationship processes in a sample of couples that were currently using the Agapé app—a light–touch relationship wellness smartphone app. The current findings suggested that the couples reported improvement across 15 of the 16 processes examined across the study period (supporting Hypothesis/Aim 1). Given that the study lacked a randomized no-treatment control group, these self-reported improvements cannot be directly attributed to use of the Agapé app as they might reflect the impact of other non-specific factors like expectancy effects, regression to the mean, self-selection, demand characteristics, or general participation effects. Despite that notable limitation, the current study represents one of the first studies in the published romantic relationship literature to attempt to model the interconnected nature of self-reported change across 16 key relationship processes and 8 relationship and individual functioning outcomes within a single, comprehensive model. Thus, multivariate, exploratory, psychological network analyses on the self-reported pre–post change observed in these couples offered some of the first (albeit correlational and preliminary) insights into how self-reported change on a wide range of relationship processes might be linked. Those analyses uncovered robust unique links between self-reported improvements on a handful of specific processes and improvement in relationship quality (supporting Hypothesis/Aim 2). The current findings further suggested patterns of interconnected change amongst a fairly comprehensive set of relationship processes (Aim 3) and highlighted several processes that appeared more centrally positioned within the broader multivariate pattern of self-reported relationship change (Aim 4). From a duocentric perspective, the current findings also offered preliminary descriptive insights into the extent to which changes in different relationship processes tended to be shared across partners versus reported more independently, with constructs such as conflict and sexual intimacy showing stronger partner-level convergence (i.e., stronger between-partner links) than most other relationship processes.

### 4.1. Implications

#### 4.1.1. The Central Linkages of Quality Time and Gratitude

Quality time spent together emerged across all analyses as a potential core (i.e., central) relationship process as increases in quality time showed strong links to corresponding increases in relationship quality. This echoes findings with positive relationship maintenance strategies (e.g., Canary & Stafford, 1992) and previous work identifying quality time as a putative mediator of relationship education (Carlson et al., 2022). From a practical standpoint, the results begin to suggest that simply prioritizing romantic relationships by setting aside time to have fun together might provide room for other processes to flourish. Alternatively, as other relationship processes improve, the results could also suggest that couples may be more willing to spend their quality time together. Further work should explore the exact functioning of quality time as it might offer a straightforward point of intervention, particularly for light–touch interventions.

Consistent with a growing body of work (e.g., Algoe et al., 2010; Barton et al., 2024; Roth et al., 2024), the current findings also highlighted the importance of cultivating gratitude within relationships. The strong unique links that emerged between changes in gratitude and changes in so many other relationship processes highlight its potential for playing a central role in self-reported relationship change. Although it would need to be explored more directly in future work, it is possible that the design of RE interventions like Agapé (which directly encourage couples to appreciate and value one another on a day-to-day basis) might help to highlight the potential importance of gratitude. The current correlated-change results (suggesting a more proximal and direct link between improvements in gratitude and improvements in relationship quality) serve as a counterpoint to recent findings highlighting indirect paths involving gratitude (e.g., Jin et al., 2024; Lambert & Fincham, 2011). Although similar indirect paths emerged for changes in gratitude in the current analyses, the robust direct link that also emerged begins to reveal the potential advantages of modeling a broader constellation of relationship processes simultaneously to clarify their unique links within the process of relationship change.

#### 4.1.2. Responsiveness as an Organizing Construct

The current analyses also identified changes in perceived partner responsiveness as a key relationship process, robustly linked to changes in relationship quality for both men and women. In fact, the unique links that emerged for perceived partner responsiveness suggested that it could potentially serve as a bridge linking changes in relationship quality to changes in many other more distal relationship processes. This finding aligns with responsiveness theory (Reis et al., 2004), which posits that our perception of our partners’ responsiveness is an integrative evaluation that “organizes” our view of many behaviors to ultimately influence our satisfaction. Thus, the current results support the possibility that perceived partner responsiveness might function as an organizing construct that could function both as (1) an active lens through which partners perceive relationship change across a wide range of relationship processes (potentially influencing those processes) and (2) as a passive/receptive summative evaluation that is influenced by relationship processes.

#### 4.1.3. Key Dynamics Linking Experiences

Notably, in the duocentric models, change in many components of men’s and women’s relationship functioning variables only demonstrated “actor links” to other within-person variables in the network. For example, while changes in quality time were quite central to both the male and female models of relationship functioning, these factors were weakly related across partners. These findings are consistent with the possibility that partners might not be describing the same shared experiences when responding to these questions. In contrast, physical affection and sexual activity both showed strong cross-partner links. Similarly, partners’ reports of conflict also showed strong cross-partner links with one another’s reports of conflict or insensitivity (though not always in expected directions). This could suggest that while these concrete behaviors may be less important in intra-individual models, they are more likely to represent a shared reality between partners. While change in conflict behaviors has been studied extensively in behavioral intervention research, further study using randomized trials is needed to understand how minimal interventions might shape these critical interactions between partners.

#### 4.1.4. Patterns of Interconnected Change

Increases in talking about one’s relationship were not directly linked to corresponding changes in relationship satisfaction for both men and women, but were instead linked by a cascade of stronger (and likely bi-directional) indirect paths. Thus, the results suggested that increases in talking about one’s relationship (outside of the app) were linked to increases in mindful awareness in that relationship, which was linked to increases in gratitude engagement and spending more quality time together, which were both robustly linked to increases in relationship satisfaction and quality. It is important to note that the current network analyses examining correlational links between change scores/trajectories were intrinsically cross-sectional in nature, leaving the directions of causality unclear. With this caveat in mind, the current network findings (like this example) offer some of the first insights into a truly multivariate understanding of self-reported relationship change. More specifically, they highlight a number of more nuanced and specific patterns of interconnected change that might be involved in relationship change and could be explored in future models and studies of relationship functioning.

### 4.2. Limitations and Future Directions

Though encouraging, the interpretation of the current findings is limited by a number of factors.

First, it is important to acknowledge that two of the authors were involved in the development of the Agapé app and are shareholders in its parent company. As a result, they have a vested interest in understanding whether the app might benefit couples and how relationship change might unfold among its users. To address this, the study was conducted and the manuscript prepared under a conflict-of-interest plan. In addition, the current analyses were conducted using pre-registered hypotheses with transparent analytic procedures (along with full disclosure of all study materials, procedures, deidentified data, and analytic syntax on osf.io: https://osf.io/vfgke, accessed on 12 July 2026). Despite those efforts, this involvement creates the potential for biases when interpreting findings. Readers should therefore interpret the current results in light of this potential conflict of interest.

Second, the current study lacked a randomized no-treatment (e.g., waitlist) condition or randomization of prompt content to control for possible placebo effects and or to disentangle demand characteristics that could have driven or amplified the changes observed across different relationship enhancement targets. As a result, it remains unclear if the multivariate relationship change and patterns of association observed in the study were due to the Agapé app’s specific impact on different relationship processes or due to other non-specific factors (e.g., expectancy effects, regression to the mean, self-selection, demand characteristics, general participation effects). Future research using randomized controlled designs is needed to determine the degree to which any of the improvements seen can be attributed to Agapé.

Third, extending the previous concern, although Agapé users experienced significant improvements on nearly all of the dimensions examined, a handful of the pre–post gains only slightly exceeded the gains typically seen in waitlist conditions of marital therapy trials (g = 0.12; Roddy et al., 2020b), suggesting those changes could possibly have been due (in part) to anticipatory effects. As a result, although the current findings can be taken as providing correlational insights into a broader multivariate pattern of relationship change, future work is needed to clearly link that change to Agapé app usage.

Fourth, the Agapé app was designed (as most RE programs are) to address a wide range of relationship processes with its prompts. It therefore represents another broadband RE program. As the targeted processes are highly interconnected and mutually activated, it becomes difficult to disentangle the “active ingredients” for such broadband RE programs. As a result, it remains unclear which of the prompts or specific processes targeted might have made the strongest contributions (if any) to the relationship improvement observed. Thus, to advance our understanding of relationship change, future research should use not only no-treatment control groups but also dismantling designs that systematically vary and contrast prompt content/targeted processes. Future research could also benefit from studying naturally occurring change (in the absence of any RE program) with multi-wave designs. This would allow for cross-lagged analyses to clarify the timing of changes in linked relationship processes that typically precede broader change in relationship quality—mapping out potential cascades of change as they organically occur.

Fifth, as the network analyses examined correlated change across the same time period, the directions of causality underlying the links observed remain unclear. In fact, it is likely that many of those links represent bidirectional (reciprocal) causal links between processes. Thus, future studies using multi-wave assessments (e.g., monthly, weekly, and daily diary studies, EMA studies) that can support longitudinal cross-lagged analyses are also needed to clarify the directions of causal influence underlying the current correlational findings.

Sixth, extending the previous point, network analyses make use of highly partialized correlations to estimate their edge weights. In fact, edge weights represent the unique correlational link between each pair of variables after controlling for (i.e., partialling out) their covariances with every other variable in the analysis. If researchers are not careful about regulating the level of collinearity amongst their variables (i.e., avoiding including variables correlated > 0.70 or 0.75 as separate variables in the same analysis), those edge weights can become excessively residualized, often shrinking toward zero and potentially becoming unstable. In the case of the current study, we pre-screened the variables in our models for excessive collinearity to prevent this issue. As a result, following current best practices (e.g., Epskamp & Fried, 2018), the simulation and bootstrapping analyses evaluating the accuracy and stability of the network models (presented in the supplemental online materials) suggested reasonable levels of stability for the current findings. However, future research could extend the current work by modeling multivariate relationship change in larger samples to ensure that the results continue to replicate.

Seventh, although 87% of couples provided follow-up data on the shorter wellness checks embedded within the Agapé app (supporting the HLM-slope analyses in Figure 4), there was substantial attrition for the longer 1-month follow-up assessment (supporting the main analyses in Figure 1, Figure 2 and Figure 3), with only 55% of couples providing that data. This rendered the main pre–post analyses dependent on slightly older respondents with greater resources. Due to this, the degree to which the current results will generalize to a broader range of couples remains unclear, as there remains a risk that the current findings might be more representative of relationship change in more stable and higher-resource couples. It will therefore be important for future studies to replicate and extend these results using incentive structures that can minimize longitudinal attrition.

Eighth, as this was a dyadic study, insisting upon dyadic participation could have discouraged the participation of racial minority, lower socio-economic status, and distressed couples (Barton et al., 2020). Future work would benefit from exploring relationship change in more diverse samples, potentially even allowing individual partners to participate without their partners to ensure the maximum generalizability of findings.

Ninth, although the hypotheses tested in this manuscript align with the pre-registered hypotheses, the proposed analyses to test those hypotheses differed slightly. We had proposed using residual change scores on the 24 dimensions assessed and then examining zero-order correlations between the processes and the outcomes as an initial test of linkages. We had further proposed following those bivariate correlations up with multiple regressions in hopes of identifying the change processes with the strongest unique links to change in relationship and individual functioning. After submitting that pre-registration, we discovered psychological network analysis and realized that it offered a far more effective and parsimonious method of examining unique links among change processes (and to support that, raw change scores might be more interpretable). Network analysis had the following clear advantages over what we had initially proposed: (1) in estimating edge weights between each pair of constructs, it controlled for all other pair-wise links in the model, (2) it would accomplish the same goal of identifying processes proximally linked to change in relationship quality, but (3) with the marked advantage of providing centrality estimates to inform those questions, and (4) offering the possibility of identifying patterns of interconnected change—something our planned analyses would be unable to do. Despite these statistical advantages, our deviation from the pre-registered plan should caution researchers to interpret our findings as exploratory and preliminary rather than confirmatory.

Tenth, although 91% of the couples in the sample were mixed-sex dyads, 36 couples were same-sex dyads—a subsample too small to support separate analyses. To ensure that the data from those couples could at least contribute in some small way to the main analyses (set up to focus on mixed-sex dyads following current guidelines; see Kennedy et al., 2015), those couples were treated as mixed-sex dyads. Future work should seek sufficient numbers of same-sex dyads to directly explore the generalizability of the current findings in those relationships.

Eleventh, the current study explored relationship change over a single month. Although the rm-ANOVA findings presented in Figure 1 suggest that modest but statistically significant change occurred over this period, that remains a fairly short-term time frame within the scope of the relationships of couples who have been together an average of 4 to 5 years. Thus, future work could extend the current preliminary findings by exploring similar patterns of multivariate change across longer time periods and potentially across major transitions in relationships (e.g., moving in together, getting engaged, getting married, having a first child). This would allow researchers to determine the degree to which the current findings might generalize or differ when examining long-term relationship change. Of course, it could also be argued that the time frame of a month could be overly long to capture the day-to-day dynamics of relationship change. Thus, future work could also extend the current findings by exploring multivariate relationship change in daily diary or experiential momentary assessment (EMA) studies, thereby clarifying the degree to which the current findings might generalize or differ when focusing on the day-to-day relationship dynamics.

Twelfth, the current findings are based entirely upon self-report data as that required the least participant burden for couples trying a self-directed RE smartphone app. As a result, the data collected is dependent upon the insight of the respondents as well as their willingness to candidly share the challenges and struggles they experience in their relationships. Of course, collecting data from both partners in each couple helped to ameliorate this concern by providing a more balanced view of each relationship in the current study. In addition, previous work has demonstrated convergent predictive validity between self-reported and observationally coded conflict behavior when predicting discord and divorce over the early years of marriage (Rogge & Bradbury, 1999), suggesting that both assessment methods may capture substantially overlapping information when modeling change in relationships. Despite these factors, future work would benefit from augmenting self-report data with observational coding of the relationship processes examined (where possible) to provide more objective assessments of key processes when modeling broader multivariate patterns of relationship change.

Finally, a large portion of the study was recruited through recent enrollees to the Agapé app and therefore reflects demographics of mobile health app users—younger, largely white, and with higher educational attainment compared to the general United States population. Although recruiting users through the organic traffic to the app likely yielded a sample representative of a population of future RE consumers, it could also have introduced selection biases into the sample—drawing couples interested in trying a light–touch RE intervention and willing to complete repeated questionnaires about their relationships. As a result, the current findings should be interpreted with caution as they might primarily provide initial insights into relationship change for couples motivated to enrich their relationships and open to sharing details on the dynamics of those relationships. Thus, future work should aim to recruit from the general population to reduce these selection effects and explore whether the interrelationships between the processes seen in this study hold for couples who are more diverse and may not already be taking steps to maintain and enhance their relationships.

## 5. Conclusions

Despite the set of limitations that qualify the interpretation of the current findings and open up a number of promising directions for future research, the current study represents one of the first studies in the published literature to attempt to model correlational links between self-reported change across 16 relationship processes and four relationship outcomes within a single model. Thus, the current findings offer novel (albeit preliminary and correlational) insights into the broader multivariate pattern of self-reported relationship change. First, it demonstrates that network analysis offers an innovative method of modeling multivariate change across a wide constellation of relationship processes and outcomes—offering initial glimpses of what could become a truly multivariate understanding of relationship change. Second, it integrates two-wave dyadic data analysis with an egocentric–duocentric network analysis perspective to deepen and enrich the questions that can be examined within longitudinal couples data. Finally, that integration of analytic approaches within pre–post data from a single-arm treatment study of couples using the Agapé app provided some of the first (tentative and correlational) insights in the literature, uncovering: (1) robust proximal links between changes on a handful of specific processes and change in relationship quality, (2) patterns of interconnected change that could be explored as candidate mechanistic cascades or pathways, and (3) a handful of processes that appeared more centrally positioned within the broader multivariate pattern of self-reported relationship change.

## Data Availability

All study materials will be made available on the first author’s osf.io profile under the “Connecting with Your Partner” project (https://osf.io/vfgke/, accessed on 12 July 2026). The SPSS syntax and HLM syntax and relevant output are also available in that same osf.io project. Finally, the data will be made available upon reasonable request within that same osf.io project.

## Supplementary Materials

The following supporting information can be downloaded at: https://www.mdpi.com/article/10.3390/bs16071182/s1, Table S1. Repeated Measures ANOVAs Testing Pre–Post Change; Figure S1. Simulations for detecting observed network edges at different sample sizes. Figure S2. Simulations for detecting stability of centrality estimates in different samples; Figure S3. Simulations for estimating precision of edge-weight estimates. Figure S4. Simulations for detecting observed networks at different sample sizes. Figure S5. Bivariate Correlations (A) and Edge Weights (B) Estimated in Men and Women; Figure S6. Bivariate Correlations (below diagonal) and Edge Weights (above diagonal) among 2-wave Pre–Post Change Scores Estimated Dyadically. Figure S7. Bivariate Correlations (below diagonal) and Edge Weights (above diagonal) among HLM Linear Slopes Estimated Dyadically.

## References

[B1-behavsci-16-01182] Acitelli L. K. (2002). Relationship awareness: Crossing the bridge between cognition and communication. Communication Theory.

[B2-behavsci-16-01182] Algoe S. B., Gable S. L., Maisel N. C. (2010). It’s the little things: Everyday gratitude as a booster shot for romantic relationships. Personal Relationships.

[B3-behavsci-16-01182] Amato P. R. (2000). The consequences of divorce for adults and children. Journal of Marriage and Family.

[B4-behavsci-16-01182] Babyak M. A. (2004). What you see may not be what you get: A brief, nontechnical introduction to overfitting in regression-type models. Psychosomatic Medicine.

[B5-behavsci-16-01182] Barton A. W., Gong Q., Guttman S., Doss B. D. (2024). Trajectories of perceived gratitude and change following relationship interventions: A randomized controlled trial with lower-income, help-seeking couples. Behavior Therapy.

[B6-behavsci-16-01182] Barton A. W., Hatch S. G., Doss B. D. (2020). If you host it online, who will (and will not) come? Individual and partner enrollment in a web-based intervention for distressed couples. Prevention Science.

[B7-behavsci-16-01182] Belli S. R., Hoekstra R. H., Pilling S., Saunders R., Stott J., Suh J. W., Ebrahimi O. V., O’Driscoll C. (2026). Using network analysis to identify processes of change in low-intensity CBT interventions for depression and anxiety disorders. Journal of Affective Disorders.

[B8-behavsci-16-01182] Braithwaite S. R., Fincham F. D. (2009). A randomized clinical trial of a computer based preventive intervention: Replication and extension of ePREP. Journal of Family Psychology.

[B9-behavsci-16-01182] Braithwaite S. R., Fincham F. D. (2011). Computer-based dissemination: A randomized clinical trial of ePREP using the actor partner interdependence model. Behaviour Research and Therapy.

[B10-behavsci-16-01182] Bringmann L. F., Elmer T., Epskamp S., Krause R. W., Schoch D., Wichers M., Wigman J. T. W., Snippe E. (2019). What do centrality measures measure in psychological networks?. Journal of Abnormal Psychology.

[B11-behavsci-16-01182] Brunner J., Austin P. C. (2009). Inflation of Type I error rate in multiple regression when independent variables are measured with error. Canadian Journal of Statistics.

[B12-behavsci-16-01182] Canary D. J., Stafford L. (1992). Relational maintenance strategies and equity in marriage. Communications Monographs.

[B13-behavsci-16-01182] Carlson R. G., Barden S. M., Locklear L., Dillman Taylor D., Carroll N. (2022). Examining quality time as a mediator of dyadic change in a randomized controlled trial of relationship education for low-income couples. Journal of Marital and Family Therapy.

[B14-behavsci-16-01182] Chen J., Chen Z. (2008). Extended Bayesian information criteria for model selection with large model spaces. Biometrika.

[B15-behavsci-16-01182] Christensen A., Atkins D. C., Berns S., Wheeler J., Baucom D. H., Simpson L. E. (2004). Traditional versus integrative behavioral couple therapy for significantly and chronically distressed married couples. Journal of Consulting and Clinical Psychology.

[B16-behavsci-16-01182] Coromina L., Guia J., Coenders G., Ferligoj A. (2008). Duocentered networks. Social Networks.

[B17-behavsci-16-01182] Crasta D., Rogge R. D., Maniaci M. R., Reis H. T. (2021). Toward an optimized measure of perceived partner responsiveness: Development and validation of the perceived responsiveness and insensitivity scale. Psychological Assessment.

[B18-behavsci-16-01182] Daks J. S., Rogge R. D., Fincham F. D. (2021). Distinguishing the correlates of being mindfully vs. mindlessly coupled: Development and validation of the Attentive Awareness in Relationships Scale (AAIRS). Mindfulness.

[B19-behavsci-16-01182] Dehle C., Larsen D., Landers J. E. (2001). Social support in marriage. The American Journal of Family Therapy.

[B20-behavsci-16-01182] Doss B. D., Benson L. A., Georgia E. J., Christensen A. (2013). Translation of integrative behavioral couple therapy to a web-based intervention. Family Process.

[B21-behavsci-16-01182] Doss B. D., Cicila L. N., Georgia E. J., Roddy M. K., Nowlan K. M., Benson L. A., Christensen A. (2016). A randomized controlled trial of the web-based OurRelationship program: Effects on relationship and individual functioning. Journal of Consulting and Clinical Psychology.

[B22-behavsci-16-01182] Doss B. D., Roddy M. K., Nowlan K. M., Rothman K., Christensen A. (2019). Maintenance of gains in relationship and individual functioning following the online OurRelationship program. Behavior Therapy.

[B23-behavsci-16-01182] Doss B. D., Thum Y. M., Sevier M., Atkins D. C., Christensen A. (2005). Improving relationships: Mechanisms of change in couple therapy. Journal of Consulting and Clinical Psychology.

[B24-behavsci-16-01182] Epskamp S., Costantini G., Haslbeck J., Isvoranu A., Cramer A. O. J., Waldorp L. J., Schmittman V. D., Borsboom D. (2021). Package ‘qgraph’ *(R package Version 1.9, Producers: Sacha Epskamp and colleages)*.

[B25-behavsci-16-01182] Epskamp S., Fried E. I. (2018). A tutorial on regularized partial correlation networks. Psychological Methods.

[B26-behavsci-16-01182] Epskamp S., Fried E. I. (2021). Package ‘bootnet’ *(R package Version 1.5, Producers: Sacha Epskamp and Eiko I. Fried)*.

[B27-behavsci-16-01182] Fentz H. N., Trillingsgaard T. (2017). Checking up on couples: A meta-analysis of the effect of assessment and feedback on marital functioning and individual mental health in couples. Journal of Marital and Family Therapy.

[B28-behavsci-16-01182] Frisch M. B., Cornell J., Villanueva M., Retzlaff P. J. (1992). Clinical validation of the quality of life inventory: A measure of life satisfaction for use in treatment planning and outcome assessment. Psychological Assessment.

[B29-behavsci-16-01182] Funk J. L., Rogge R. D. (2007). Testing the ruler with item response theory: Increasing precision of measurement for relationship satisfaction with the couples satisfaction index. Journal of Family Psychology.

[B30-behavsci-16-01182] Halford W. K., Moore E., Wilson K. L., Farrugia C., Dyer C. (2004). Benefits of flexible delivery relationship education: An evaluation of the couple CARE program. Family Relations.

[B31-behavsci-16-01182] Hawrilenko M., Gray T. D., Córdova J. V. (2016). The heart of change: Acceptance and intimacy mediate treatment response in a brief couples intervention. Journal of Family Psychology.

[B32-behavsci-16-01182] Jin L., Zhu T., Wang Y. (2024). Relationship power attenuated the effects of gratitude on perceived partner responsiveness and satisfaction in romantic relationships. Scientific Reports.

[B33-behavsci-16-01182] Karney B. R., Bradbury T. N. (1995). The longitudinal course of marital quality and stability: A review of theory, methods, and research. Psychological Bulletin.

[B34-behavsci-16-01182] Kazdin A. E. (2009). Understanding how and why psychotherapy leads to change. Psychotherapy Research.

[B35-behavsci-16-01182] Kennedy D. P., Jackson G. L., Green H. D., Bradbury T. N., Karney B. R. (2015). The analysis of duocentric social networks: A primer. Journal of Marriage and Family.

[B36-behavsci-16-01182] Kroenke K., Spitzer R. L., Williams J. B. (2001). The PHQ-9: Validity of a brief depression severity measure. Journal of General Internal Medicine.

[B37-behavsci-16-01182] Lambert N. M., Fincham F. D. (2011). Expressing gratitude to a partner leads to more relationship maintenance behavior. Emotion.

[B38-behavsci-16-01182] Le Y., Roddy M. K., Hatch S. G., Doss B. D. (2020). Mechanisms of improvements and maintenance in online relationship programs for distressed low-income couples. Journal of Consulting and Clinical Psychology.

[B39-behavsci-16-01182] Markman H. J., Hawkins A. J., Stanley S. M., Halford W. K., Rhoades G. (2022). Helping couples achieve relationship success: A decade of progress in couple relationship education research and practice, 2010–2019. Journal of Marital and Family Therapy.

[B40-behavsci-16-01182] Markman H. J., Stanley S., Blumberg S. L. (1994). Fighting for your marriage.

[B41-behavsci-16-01182] Owen J., Rhoades G. K., Stanley S. M., Markman H. J. (2011). The revised commitment inventory: Psychometrics and use with unmarried couples. Journal of Family Issues.

[B42-behavsci-16-01182] Pollard A. E., Rogge R. D. (2022). Love in the time of COVID-19: A multi-wave study examining the salience of sexual and relationship health during the COVID-19 pandemic. Archives of Sexual Behavior.

[B43-behavsci-16-01182] Rasmussen B. D., Daks J. S., Rogge R. D. (2026). Thanks, unspoken: The critical roles of feeling grateful and expressing gratitude in romantic relationships.

[B44-behavsci-16-01182] Reis H. T., Clark M. S., Holmes J. G. (2004). Perceived partner responsiveness as an organizing construct in the study of intimacy and closeness. Handbook of closeness and intimacy.

[B45-behavsci-16-01182] Roddy M. K., Stamatis C. A., Rothman K., Doss B. D. (2020a). Mechanisms of change in a brief, online relationship intervention. Journal of Family Psychology.

[B46-behavsci-16-01182] Roddy M. K., Walsh L. M., Rothman K., Hatch S. G., Doss B. D. (2020b). Meta-analysis of couple therapy: Effects across outcomes, designs, timeframes, and other moderators. Journal of Consulting and Clinical Psychology.

[B47-behavsci-16-01182] Rodrigues A. E. (2010). Examining moderating and mediating effects of aversive behavior in romantic relationships *(Pub. No. 3430810)*. Doctoral dissertation.

[B48-behavsci-16-01182] Rogge R. D., Bradbury T. N. (1999). Till violence does us part: The differing roles of communication and aggression in predicting adverse marital outcomes. Journal of Consulting and Clinical Psychology.

[B49-behavsci-16-01182] Rogge R. D., Cobb R. J., Lawrence E., Johnson M. D., Bradbury T. N. (2013). Is skills training necessary for the primary prevention of marital distress and dissolution? A 3-year experimental study of three interventions. Journal of Consulting and Clinical Psychology.

[B50-behavsci-16-01182] Rogge R. D., Fincham F. D., Crasta D., Maniaci M. R. (2017). Positive and negative evaluation of relationships: Development and validation of the Positive–Negative Relationship Quality (PN-RQ) scale. Psychological Assessment.

[B51-behavsci-16-01182] Rogge R. D., Macri J. A., Okwudili K. (2024). Connection at your fingertips: A first look at the Agapé app’s contributions to healthy relationships. Journal of Family Psychology.

[B52-behavsci-16-01182] Rolffs J. L., Rogge R. D., Knee C. R., Reis H. T. (2016). Brief interventions to strengthen relationships and prevent dissolution. Positive Approaches to optimal relationship development.

[B53-behavsci-16-01182] Roth M., Good N., Ledermann T., Landolt S. A., Weitkamp K., Bodenmann G. (2024). Building happier bonds: Gratitude as a mediator between dyadic coping and relationship satisfaction in romantic couples. Frontiers in Psychology.

[B54-behavsci-16-01182] Rothman K., Roddy M. K., Doss B. D. (2019). Completion of a stand-alone versus coach-supported trial of a web-based program for distressed relationships. Family Relations.

[B55-behavsci-16-01182] Schramm D. G. (2006). Individual and social costs of divorce in Utah. Journal of Family and Economic Issues.

[B56-behavsci-16-01182] Shaw A. M., Rogge R. D. (2016). Evaluating and refining the construct of sexual quality with item response theory: Development of the Quality of Sex Inventory. Archives of Sexual Behavior.

[B57-behavsci-16-01182] Stanton S. C., Chan A. P. S., Gazder T. (2021). Mindfulness, perceived partner responsiveness, and relationship quality: A dyadic longitudinal mediation model. Journal of Social and Personal Relationships.

[B58-behavsci-16-01182] Sullivan K. T., Pasch L. A., Eldridge K. A., Bradbury T. N. (1998). Social support in marriage: Translating research into practical applications for clinicians. The Family Journal.

[B59-behavsci-16-01182] Tibshirani R. (1996). Regression shrinkage and selection via the LASSO. Journal of the Royal Statistical Society. Series B (Methodological).

[B60-behavsci-16-01182] van Gemert-Pijnen J. E., Nijland N., van Limburg M., Ossebaard H. C., Kelders S. M., Eysenbach G., Seydel E. R. (2011). A holistic framework to improve the uptake and impact of eHealth technologies. Journal of Medical Internet Research.

[B61-behavsci-16-01182] Watson D., Clark L. A., Weber K., Assenheimer J. S., Strauss M. E., McCormick R. A. (1995). Testing a tripartite model: II. Exploring the symptom structure of anxiety and depression in student, adult, and patient samples. Journal of Abnormal Psychology.

