# Supplementary material for: A Blueprint for Connection: Mapping Interconnected Patterns of Relationship Change in Couples Using the Agapé App
Source: Behav Sci (Basel). 2026 Jul 13;16(7):1182. doi: 10.3390/bs16071182 (PMC13405681; doi:10.3390/bs16071182)
Supplement: Supplementary file 1 [file behavsci-16-01182-s001.zip › behavsci-4288782-supplementary.pdf]

Supplemental Online Table S1. *Repeated Measures ANOVAs Testing Pre-Post Change*

		Dyadic RM ANOVAs			Results within Male Partners					Results within Female Partners				
CLASS OF CONSTRUCTS		F-statistics			Raw Change scores			RM ANOVA		Raw Change scores			RM ANOVA	
-- Construct	Range	ME_{time}	ME_{sex}	INT	M	(SD)	d_{rm}	*F*	*p*	M	(SD)	d_{rm}	*F*	*p*
RELATIONSHIP FUNCTIONING														
-- Relationship satisfaction	0 to 41	**23.85**	0.18	1.70	1.99	6.59	**0.39**	**14.0**	**<.001**	2.99	7.69	**0.43**	**29.5**	**<.001**
-- Dedication to relationship	1 to 6	**9.04**	0.09	0.50	0.12	0.66	**0.22**	**4.6**	**.033**	0.14	0.76	**0.21**	**7.8**	**.006**
-- Positive rel qualities	1 to 6	**3.95**	1.89	0.08	0.09	1.20	0.09	0.9	.352	0.21	1.21	**0.21**	**5.7**	**.018**
-- Negative rel qualities	1 to 6	**28.22**	0.01	0.20	-0.27	0.61	**-0.50**	**31.3**	**<.001**	-0.26	0.81	**-0.29**	**24.2**	**<.001**
INDIVIDUAL FUNCTIONING														
-- Vitality	1 to 6	**6.20**	**13.63**	2.15	0.32	1.21	**0.31**	**8.7**	**.004**	0.21	1.48	**0.14**	**4.2**	**.041**
-- Quality of life	1 to 6	**6.54**	0.03	0.00	0.18	0.82	**0.27**	**6.3**	**.013**	0.16	0.95	**0.19**	**8.3**	**.004**
-- Psychological distress	1 to 6	**17.02**	1.25	0.50	-0.39	1.22	**-0.33**	**14.9**	**<.001**	-0.40	1.41	**-0.30**	**14.9**	**<.001**
-- Depressive symptoms	0 to 27	**19.53**	0.31	0.41	-1.22	3.60	**-0.49**	**15.5**	**<.001**	-1.41	4.49	**-0.42**	**18.7**	**<.001**

		Dyadic RM ANOVAs				Results in Male Partners					Results in Female Partners				
CLASS OF CONSTRUCTS		F-statistics				Descriptives			RM ANOVA		Descriptives			RM ANOVA	
-- Construct	Range	ME_{time}	ME_{sex}	INT		M	(SD)	d_{rm}	F	p	M	(SD)	d_{rm}	F	p
REL MAINTENANCE PROCESSES															
-- Learning about partner	1 to 6	**37.45**	0.24	1.07	Pre	3.8	1.3	**0.41**	**21.7**	**<.001**	3.6	1.2	**0.40**	**42.7**	**<.001**
					Post	4.3	1.2				4.3	1.3			
-- Reflecting on own behavior	1 to 6	**16.40**	0.24	0.19	Pre	4.2	1.1	**0.26**	**9.5**	**.002**	4.1	1.1	**0.32**	**22.5**	**<.001**
					Post	4.5	1.0				4.5	0.9			
-- Talking about your rel	1 to 6	**25.44**	0.25	1.10	Pre	3.6	1.2	**0.46**	**22.9**	**<.001**	3.6	1.2	**0.43**	**28.3**	**<.001**
					Post	4.0	1.1				4.0	1.2			
-- Quality time together	1 to 6	**12.41**	0.06	0.04	Pre	4.3	1.0	**0.29**	**7.5**	**.007**	4.3	1.0	**0.38**	**19.9**	**<.001**
					Post	4.5	1.0				4.6	1.0			
-- Telling, "I love you"	1 to 6	**4.49**	0.00	0.99	Pre	4.9	1.4	0.13	1.5	.226	4.9	1.5	**0.34**	**9.5**	**.002**
					Post	5.0	1.3				5.1	1.3			
CONNECTIVE REL PROCESSES															
-- Emotional support	1 to 6	**44.14**	0.07	0.09	Pre	4.3	1.0	**0.53**	**27.0**	**<.001**	4.2	1.1	**0.67**	**52.5**	**<.001**
					Post	4.6	1.0				4.6	1.1			
-- Attentive rel awareness	1 to 6	**19.08**	0.20	0.00	Pre	4.2	1.1	**0.30**	**10.8**	**.001**	4.2	1.2	**0.33**	**20.2**	**<.001**
					Post	4.5	1.0				4.6	1.0			
-- Gratitude toward partner	1 to 6	**33.45**	0.04	2.62	Pre	4.4	1.1	**0.39**	**12.1**	**.001**	4.4	1.2	**0.49**	**34.9**	**<.001**
					Post	4.7	1.0				4.8	1.1			
-- PP responsiveness	1 to 6	**10.12**	0.23	***3.85***	Pre	4.3	1.2	0.07	0.5	.502	4.2	1.2	**0.28**	**15.3**	**<.001**
					Post	4.4	1.2				4.6	1.1			
SEXUAL FUNCTIONING															
-- Sexual activity	1 to 6	**12.15**	0.18	0.31	Pre	1.5	0.8	**0.23**	**8.1**	**.005**	1.5	0.9	***0.12***	***3.4***	***0.065***
					Post	1.7	0.8				1.7	0.8			
-- Physical affection	1 to 6	***3.36***	0.15	***2.94***	Pre	3.5	1.5	0.04	0.2	.655	3.4	1.6	**0.18**	**6.5**	**.012**
					Post	3.6	1.6				3.7	1.7			
-- Sexual satisfaction	1 to 6	0.91	0.57	0.13	Pre	3.7	1.9	0.01	0.0	.910	3.6	1.9	**0.20**	**6.2**	**0.014**
					Post	3.7	1.7				3.9	1.8			
DETACHING REL PROCESSES															
-- Failing to express gratitude	1 to 6	***70.07***	0.33	***3.55***	Pre	2.6	1.0	**-0.52**	**33.2**	**<.001**	2.9	1.3	**-0.60**	**71.3**	**<.001**
					Post	2.2	1.0				2.1	1.0			
-- Negative conflict behavior	1 to 6	**39.64**	1.35	0.22	Pre	1.4	0.4	**-0.42**	**26.9**	**<.001**	1.4	0.5	**-0.45**	**45.6**	**<.001**
					Post	1.2	0.3				1.2	0.4			
-- Distraction from relationship	1 to 6	**23.34**	1.15	0.01	Pre	2.3	1.1	**-0.29**	**12.0**	**.001**	2.3	1.2	**-0.41**	**31.8**	**<.001**
					Post	2.0	1.0				1.8	0.9			
-- PP insensitivity	1 to 6	**4.44**	0.39	**4.06**	Pre	1.7	0.8	-0.08	0.8	.362	1.7	0.9	**-0.24**	**11.2**	**.001**
					Post	1.6	0.8				1.5	0.8			

NOTE: RM ANOVA = the results of within-person repeated-measures ANOVAs; ME_{time} = within person main effects for time (pre-post change); ME_{sex} = within couple main effect for sex (male vs female partner); INT = time by sex interaction suggesting differential change over time by sex; d_{rm} = repeated-measures Cohen's d; rel = relationship; behv = behavior; PP = perceived partner; Pre = baseline assessment; Post = 1-month assessment. To facilitate interpretation, effects significant at p < .05 have been bolded and marginally significant effects (p < .10) have been bolded and italicized.

Supplemental Online Figure S1. *Simulations for detecting observed network edges at different sample sizes.*

Correlations of edge weights between observed and simulated networks along with the sensitivity and specificity for detecting those edges at different sample sizes.

A) From simulations of Pre-Post model in MEN

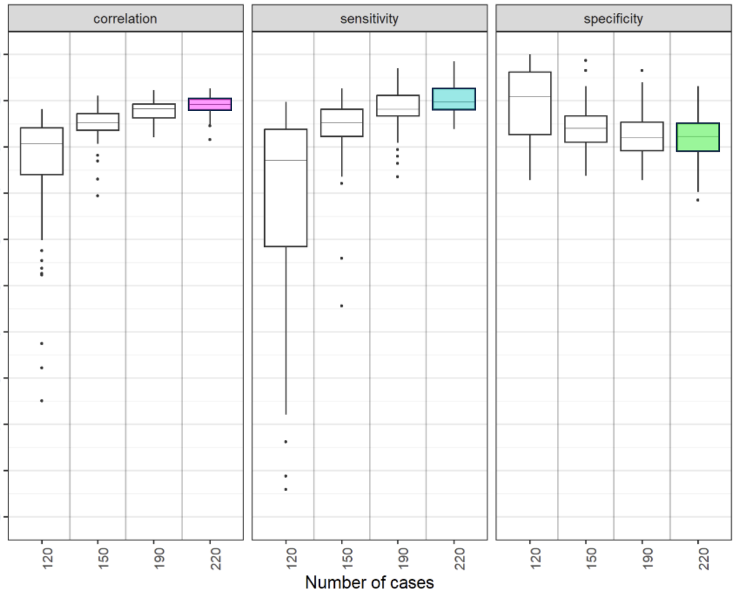

B) From simulations of the Pre-Post model in WOMEN

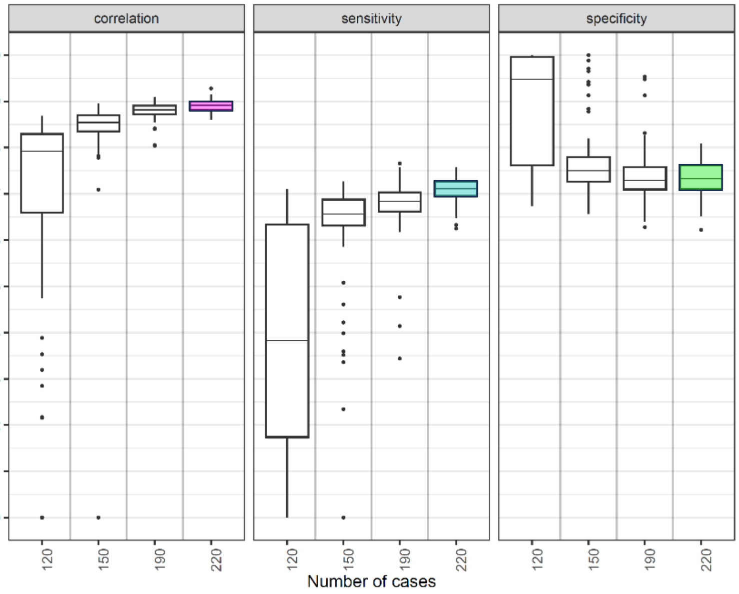

C) From simulations of the DYADIC Pre-Post model

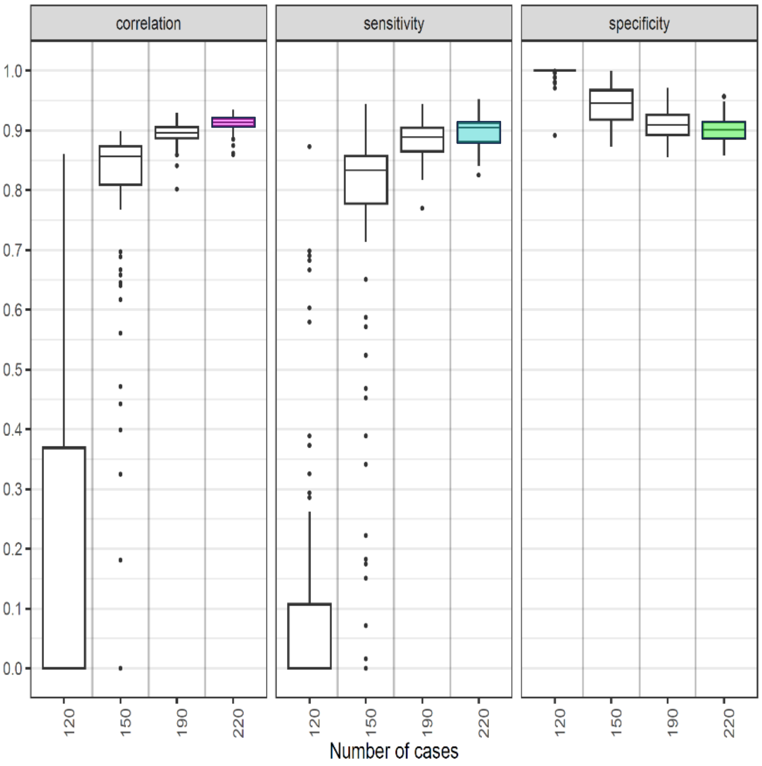

D) From simulations of the DYADIC HLM-slope model

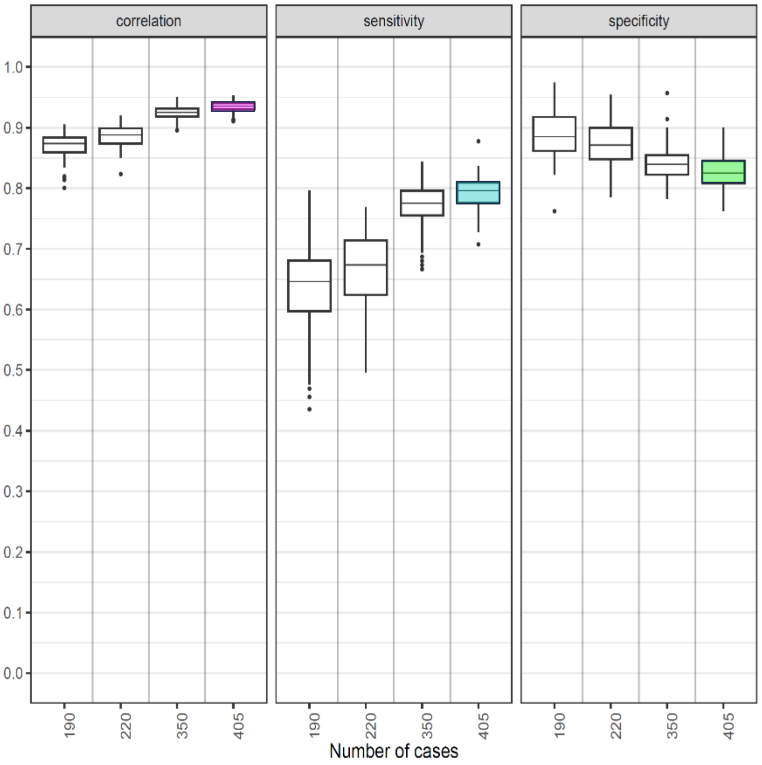

Supplemental Online Figure S2. *Simulations for detecting stability of centrality estimates in different sample sizes.*

Correlations of centrality indices between observed and simulated networks at different sample sizes.

A) From simulations of Pre-Post model in MEN

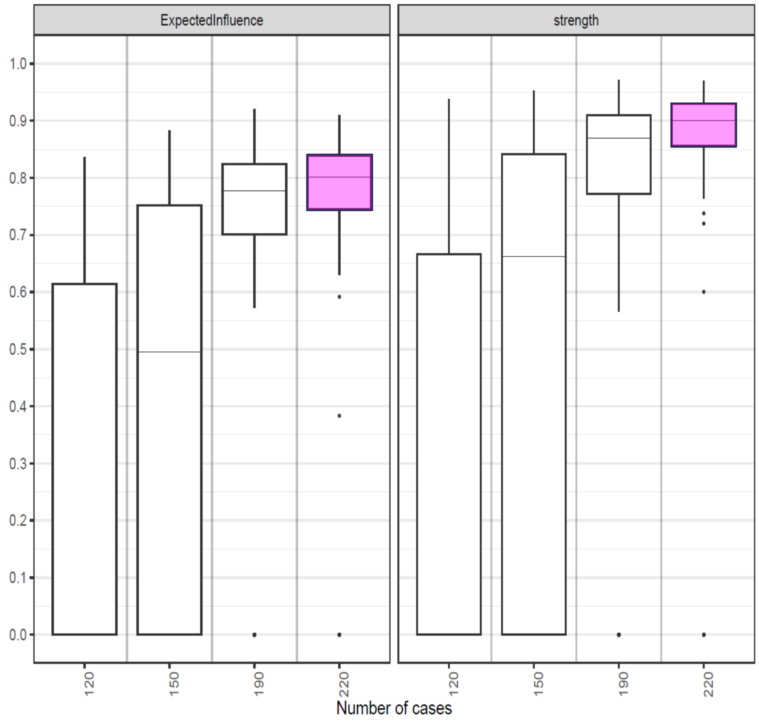

B) From simulations of the Pre-Post model in WOMEN

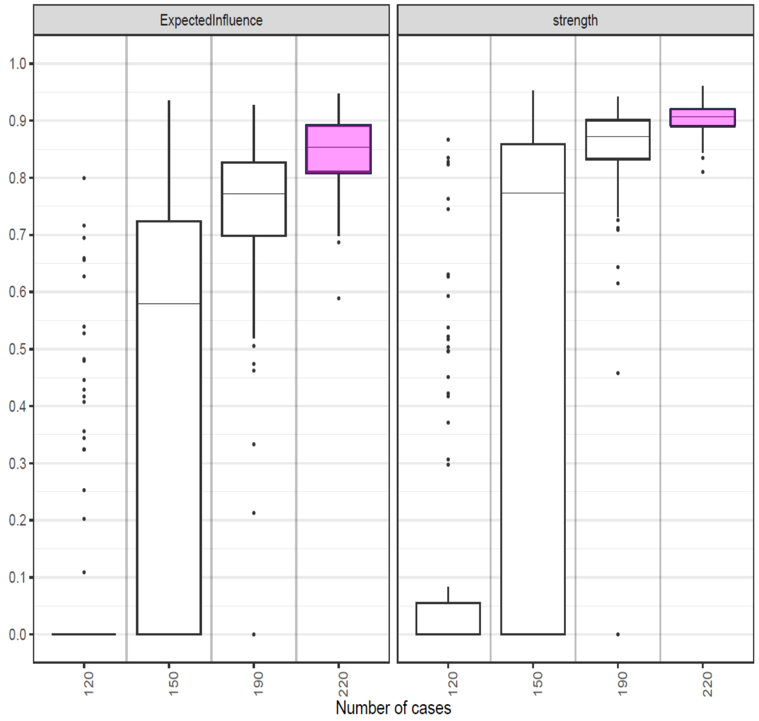

C) From simulations of the DYADIC Pre-Post model

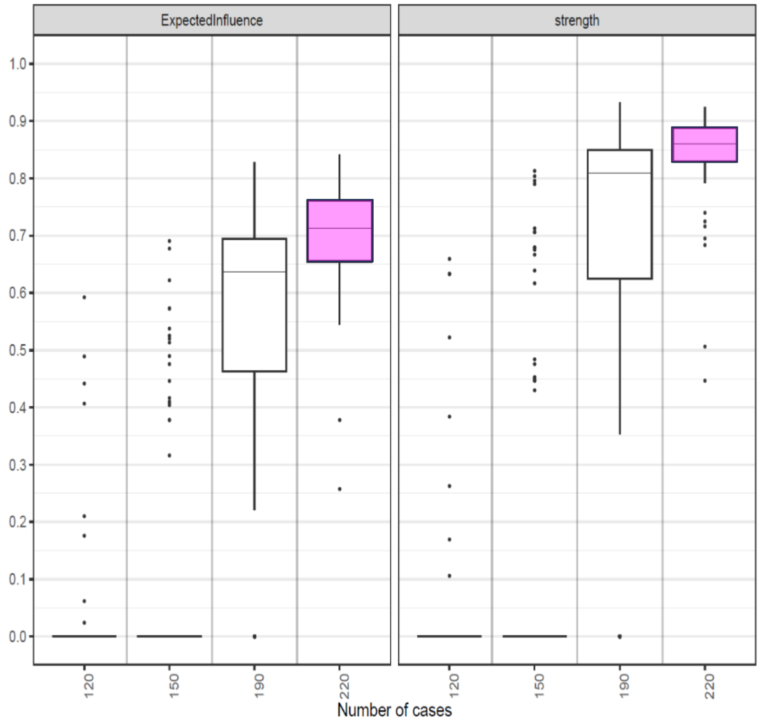

D) From simulations of the DYADIC HLM-slope model

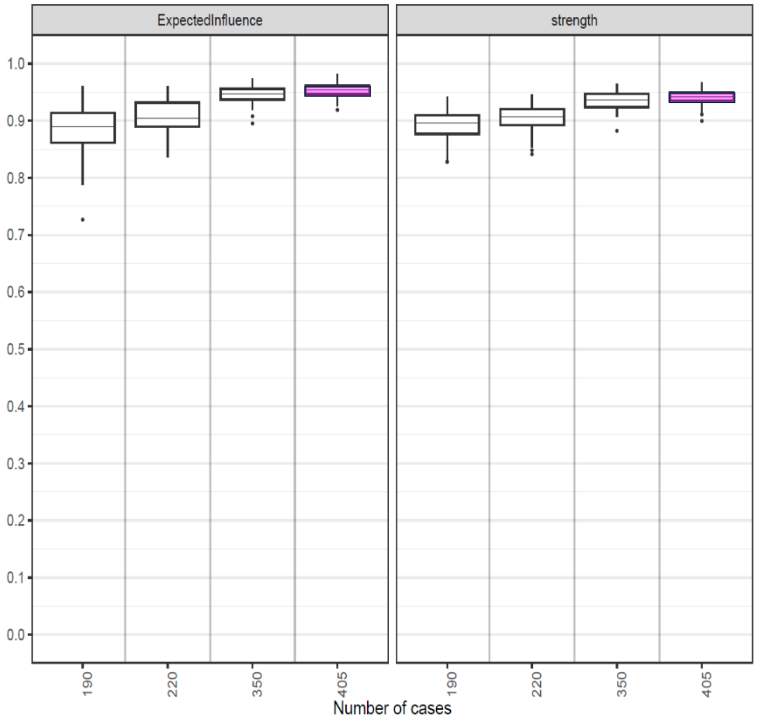

Supplemental Online Figure S3. *Simulations for estimating precision of edge-weight estimates.*

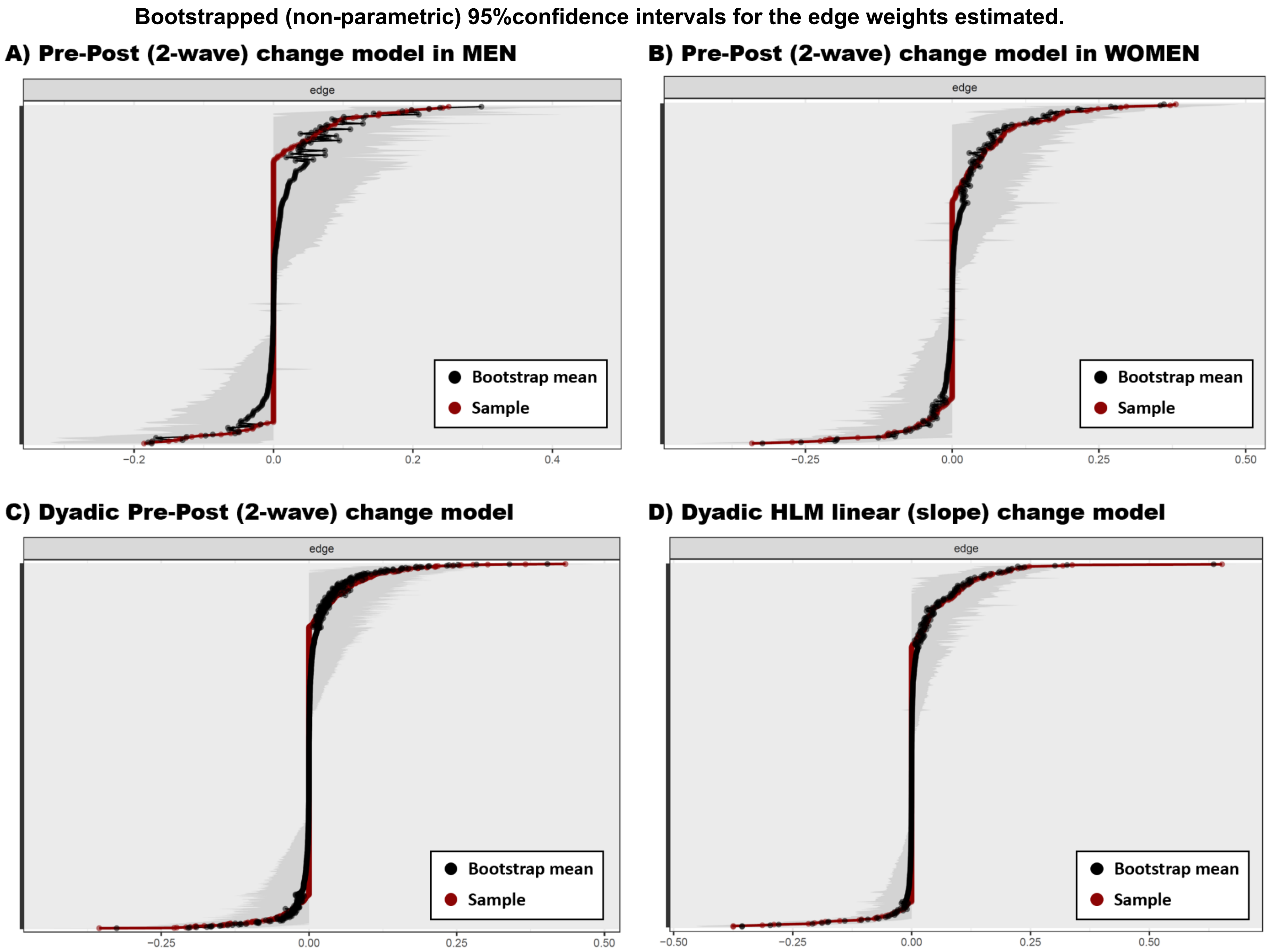

Supplemental Online Figure S4. *Simulations for detecting observed networks at different sample sizes.*

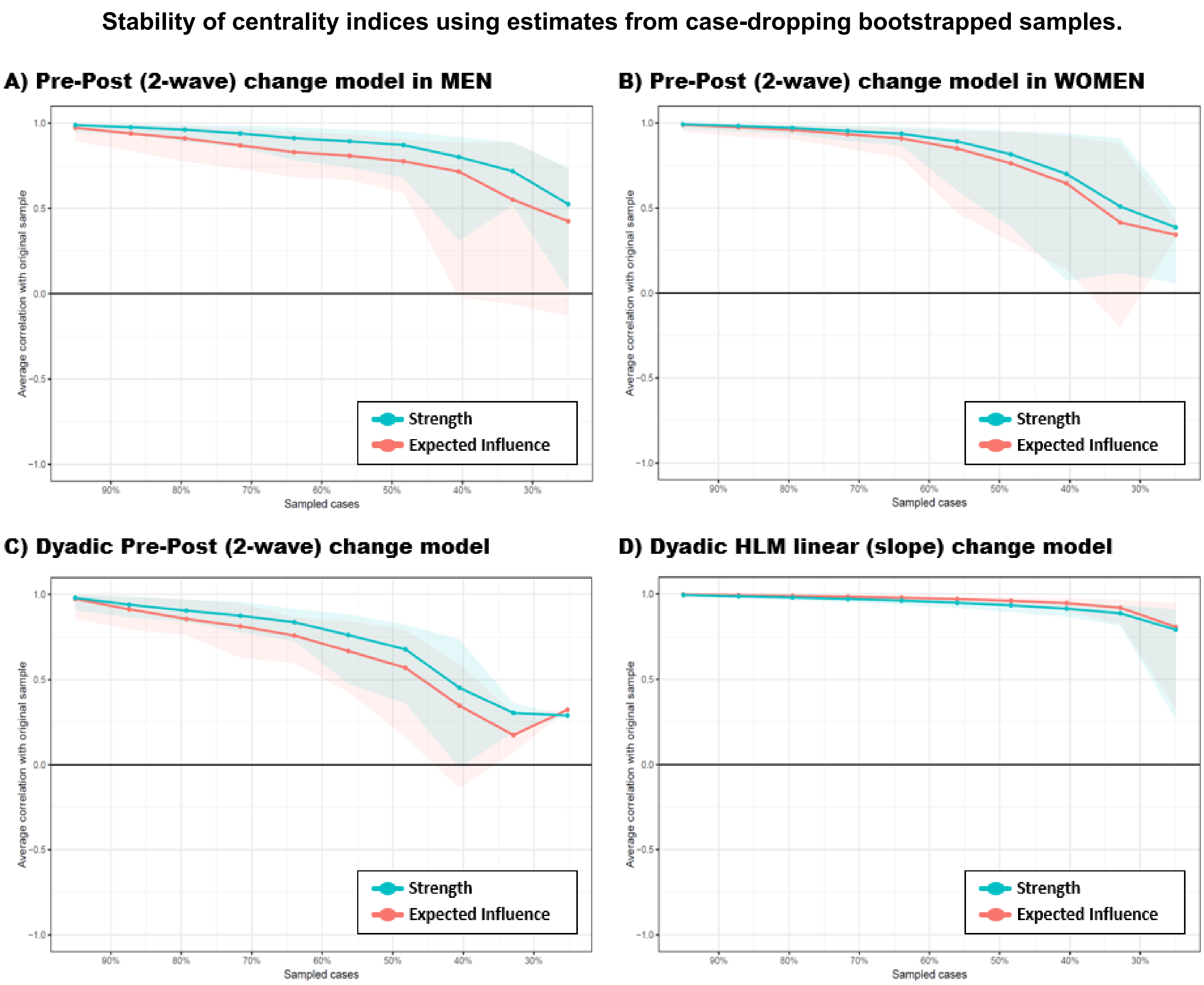

Supplemental Online Figure S5. *Bivariate Correlations (A) and Edge Weights (B) Estimated in Men and Women Separately. NOTE: The edge weights represent unique associations between each pair of variables (nodes). Specifically, they are partial correlations between each pair of variables controlling for all other variables in the model.*

A)

Bi-variate Correlations among 2-Wave Raw Change Scores in Men (above diagonal) and Women (below diagonal)

CLASS OF VARIABLES		REL FUNCTION					IND FUNCTION				REL MAINTENANCE					CONNECTIVE				SEX FN			DETACHING			
#	Specific variable	0	1	2	3	4	5	6	7	8	9	10	11	12	13	14	15	16	17	18	19	20	21	22	23	24
RELATIONSHIP FUNCTIONING																										
0	Baseline relationship satisfaction		-.35	-.22	-.21	.36	-.05	-.04	.09	.14	-.09	-.08	-.03	-.26	-.13	-.17	-.17	-.31	-.30	-.11	-.26	-.19	.03	.27	.27	.05
1	Δ Relationship satisfaction	-.49		.38	.67	-.58	.38	.20	-.18	-.25	.22	.19	.11	.69	.39	.40	.37	.64	.55	.14	.32	.37	-.24	-.19	-.30	-.45
2	Δ Dedication to relationship	-.20	.42		.29	-.32	.18	.04	.03	-.10	.07	.29	.20	.34	.23	.19	.27	.38	.30	.03	.15	.17	-.17	-.18	-.20	-.15
3	Δ Positive relationship qualities	-.30	.69	.39		-.50	.35	.29	-.22	-.21	.30	.22	.14	.67	.34	.38	.40	.55	.47	.14	.29	.31	-.41	-.07	-.31	-.31
4	Δ Negative relationship qualities	.26	-.57	-.36	-.52		-.25	-.16	.26	.21	-.18	-.06	-.07	-.37	-.25	-.32	-.19	-.47	-.43	-.06	-.22	-.22	.25	.37	.35	.31
INDIVIDUAL FUNCTIONING																										
5	Δ Vitality	-.06	.28	.14	.32	-.32		.36	-.48	-.46	.17	.10	.14	.29	.09	.26	.22	.21	.23	.01	.21	.27	-.10	-.15	-.17	-.27
6	Δ Quality of life	-.08	.16	.18	.32	-.18	.42		-.42	-.23	.21	.15	.23	.27	.17	.18	.16	.13	.17	.02	.27	.27	-.07	-.03	-.19	-.17
7	Δ Psychological distress	.30	-.39	-.15	-.36	.42	-.59	-.26		.55	.04	-.03	-.09	-.13	-.02	-.17	-.13	-.06	-.07	.08	-.16	-.12	.08	.15	.20	.19
8	Δ Depressive symptoms	.11	-.19	-.16	-.30	.34	-.54	-.33	.63		-.05	-.05	-.15	-.19	-.16	-.19	-.18	-.14	-.27	.01	-.18	-.20	.14	.06	.25	.15
RELATIONSHIP MAINTENANCE PROCESSES																										
9	Δ Learning new things about partner	-.15	.38	.27	.43	-.34	.17	.22	-.21	-.11		.33	.29	.37	.20	.22	.30	.29	.25	.07	.12	.17	-.23	.05	-.24	-.06
10	Δ Reflecting on your own behavior in rel	.02	.18	.04	.21	-.18	.12	.20	-.08	-.05	.33		.21	.43	.12	.22	.29	.38	.31	-.02	.02	.08	-.19	-.06	-.24	-.13
11	Δ Talking about your rel	.01	.19	.10	.32	-.23	.26	.15	-.13	-.21	.44	.46		.24	.26	.18	.37	.28	.28	.12	.26	.24	-.13	-.03	-.29	-.06
12	Δ Quality time together	-.31	.58	.28	.57	-.52	.26	.30	-.29	-.27	.49	.39	.39		.32	.37	.51	.56	.51	.16	.35	.35	-.30	-.13	-.36	-.24
13	Δ Telling each other, "I love you"	-.09	.30	.19	.30	-.33	.03	.12	-.04	-.07	.20	.09	.21	.29		.08	.31	.23	.31	.09	.20	.15	-.16	-.11	-.31	-.17
CONNECTIVE RELATIONSHIP PROCESSES																										
14	Δ Emotional support	-.09	.40	.30	.40	-.30	.31	.30	-.23	-.13	.27	.24	.16	.35	.17		.29	.33	.40	-.11	.05	.12	-.20	-.25	-.28	-.30
15	Δ Attentive awareness in relationship	-.14	.39	.26	.38	-.36	.33	.28	-.28	-.27	.28	.33	.45	.39	.11	.24		.51	.40	.09	.20	.24	-.35	-.14	-.47	-.15
16	Δ Gratitude toward partner	-.25	.53	.36	.46	-.49	.32	.23	-.27	-.18	.38	.36	.41	.57	.23	.38	.58		.50	.08	.20	.23	-.44	-.13	-.37	-.32
17	Δ Perceived partner responsiveness	-.31	.60	.22	.56	-.48	.26	.15	-.24	-.17	.37	.24	.26	.50	.23	.32	.36	.47		.10	.21	.28	-.35	-.14	-.28	-.36
SEXUAL FUNCTIONING																										
18	Δ Frequency of sexual activity	-.15	.20	.05	.25	-.02	.21	.21	-.11	-.11	.06	-.03	.14	.22	.19	.09	.11	.12	.08		.44	.31	.01	.00	-.03	.01
19	Δ Frequency of physical affection	-.23	.26	.01	.30	-.22	.23	.14	-.24	-.19	.08	.05	.24	.30	.19	.04	.26	.26	.15	.55		.47	-.12	-.07	-.17	-.06
20	Δ Sexual satisfaction	-.28	.53	.19	.42	-.41	.37	.25	-.29	-.12	.34	.32	.41	.54	.19	.31	.44	.47	.40	.40	.38		-.02	-.06	-.19	-.21
DETACHING RELATIONSHIP PROCESSES																										
21	Δ Failing to express gratitude	.11	-.26	-.12	-.23	.30	-.27	-.08	.31	.24	-.26	-.17	-.21	-.28	-.13	-.15	-.36	-.39	-.21	.09	-.17	-.20		.12	.27	.05
22	Δ Negative conflict behavior	.14	-.35	-.27	-.31	.45	-.22	-.24	.18	.15	-.28	-.27	-.20	-.32	-.21	-.29	-.29	-.44	-.31	.07	-.04	-.15	.19		.35	.11
23	Δ Distraction from relationship	.17	-.34	-.20	-.37	.33	-.27	-.06	.24	.19	-.26	-.31	-.42	-.36	-.10	-.24	-.61	-.45	-.28	-.06	-.15	-.41	.46	.26		.22
24	Δ Perceived partner insensitivity	.30	-.44	-.22	-.36	.57	-.13	-.16	.19	.12	-.27	-.15	-.15	-.31	-.25	-.23	-.21	-.32	-.50	.00	-.18	-.26	.11	.31	.05	

NOTE: Δ = pre-post change (raw); rel = relationship; IND = individual; CONNECTIVE = connective relationship processes; SEX FN = sexual functioning; DETACHING = detaching relationship processes. All corrs |.16| or higher sig at p < .05. Corrs |.30| or greater have been bolded and cells have been shaded from red (negative correlation) to blue (positive correlation) to facilitate interpretation.

B)

Edge Weights (partial correlations) among 2-Wave Raw Change Scores from separate network analyses in Men (above diagonal) and in Women (below diagonal)

CLASS OF VARIABLES		REL FUNCTION					IND FUNCTION				REL MAINTENANCE					CONNECTIVE				SEX FN			DETACHING			
#	Specific variable	0	1	2	3	4	5	6	7	8	9	10	11	12	13	14	15	16	17	18	19	20	21	22	23	24
RELATIONSHIP FUNCTIONING																										
0	Baseline relationship satisfaction	---	-.05			.06																				
1	Δ Relationship satisfaction	-.22	---	.06	.19	-.19	.08							.22	.08	.06		.20	.12		.01	.06				-.14
2	Δ Dedication to relationship		-.11	---		-.01						.00		.01				.07								
3	Δ Positive relationship qualities		.29	-.07	---	-.10	.03	.01						.24	.03	.04		.09	.04				-.08			
4	Δ Negative relationship qualities		.10	-.06	.08	---												-.06	-.05					.09	.04	
INDIVIDUAL FUNCTIONING																										
5	Δ Vitality						---	.06	-.15	-.13																
6	Δ Quality of life				.05		.19	---	-.13																	
7	Δ Psychological distress	.06	-.05	.11	-.02		-.27		---	.25																
8	Δ Depressive symptoms			.04	-.01		-.21	-.07	.37	---																
RELATIONSHIP MAINTENANCE PROCESSES																										
9	Δ Learning new things about partner	.00	.08	-.11	.13			.04			---	.05	.02	.08			.00									
10	Δ Reflecting on your own behavior in rel		.04	-.10	.05						.05	---		.11				.06								
11	Δ Talking about your rel						.01				.01	.01	---				.09		.00							
12	Δ Quality time together							.00			.11		.24	---		.02	.15	.07	.10		.05	.03			-.02	
13	Δ Telling each other, "I love you"				.10	.02					.18		.18	.05	---		.01		.01						-.03	
CONNECTIVE RELATIONSHIP PROCESSES																										
14	Δ Emotional support		.06		.09	.08	.07	.09			.02			.02	.01	---			.09							
15	Δ Attentive awareness in relationship			.00	.01		.05	.03		-.01			.11	.02			---	.15	.04				-.03		-.17	
16	Δ Gratitude toward partner		.07	-.06		.07	.00				.18		.05	.04	.00	.08	.25	---	.10				-.12		-.03	
17	Δ Perceived partner responsiveness		.19	-.03	.15						.07				.06	.01	.02	.08	---				-.02			-.04
SEXUAL FUNCTIONING																										
18	Δ Frequency of sexual activity			.17		-.03		-.05				-.01		-.05	-.04	-.06		-.16	.00	---	.15	.01				
19	Δ Frequency of physical affection			.01	-.06								-.11	-.03			-.34	-.02		.00	---	.18				
20	Δ Sexual satisfaction			.03			-.02		.09						-.04		-.01	-.12			.23	---				
DETACHING RELATIONSHIP PROCESSES																										
21	Δ Failing to express gratitude	.06	-.03	.30								-.03							-.22	.03			---			
22	Δ Negative conflict behavior	-.03			.04		.01		-.03		.03	.01	.02				.02							---	.07	
23	Δ Distraction from relationship						.00	.05				.03								.03		.04		.38	---	
24	Δ Perceived partner insensitivity		.15	-.01			.09				.16		.10	.02		.02	.08	.05	.02		-.08			.09	.17	---

NOTE: Δ = pre-post change (raw); rel = relationship; IND = individual; CONNECTIVE = connective relationship processes; SEX FN = sexual functioning; DETACHING = detaching relationship processes. Edge weights |.20| or greater are bolded and cells have been shaded from red (negative edge weight) to blue (positive edge weight) to facilitate interpretation.

Supplemental Online Figure S6. *Bivariate Correlations (below diagonal) and Edge Weights (above diagonal) among 2-wave Pre-Post Change Scores Estimated Dyadically. NOTE: Edge weights represent unique associations between each pair of variables (nodes). Specifically, they are partial correlations between each pair of variables controlling for all other variables in the model.*

CORRELATIONS (below diagonal) AND EDGE-WEIGHTS (above diagonal) AMONG 2-WAVE CHANGE SCORES

GENDER — CHANGE IN MEN (columns)

CLASS OF VARIABLES		REL FUNCTION					IND FUNCTION				REL MAINTENANCE					CONNECTIVE				SEX FN			DETACHING			
#	Specific variable	0	1	2	3	4	5	6	7	8	9	10	11	12	13	14	15	16	17	18	19	20	21	22	23	24
	**CHANGE IN MEN**																									
	**RELATIONSHIP FUNCTIONING**																									
0	Baseline rel satisfaction					.09													-.05		-.03			.02	.04	
1	Δ Relationship satisfaction	-.35		.04	.14	-.14	.10							.24	.09	.01		.21	.06		.00	.04				-.13
2	Δ Dedication to relationship	-.22	.38									.05		.04			.02	.02	.02							
3	Δ Positive rel qualities	-.21	.67	.29		-.10	.06	.08			.04			.26	.04	.07		.12	.06			.02	-.10			-.05
4	Δ Negative rel qualities	.36	-.58	-.32	-.50				.04	.00						-.03		-.04	-.10		-.03			.09	.07	.06
	**INDIVIDUAL FUNCTIONING**																									
5	Δ Vitality	-.05	.38	.18	.35	-.25		.11	-.13	-.19												.01				
6	Δ Quality of life	-.04	.20	.04	.29	-.16	.36		-.15												.06	.01				
7	Δ Psychological distress	.09	-.18	.03	-.22	.26	-.48	-.42		.32																
8	Δ Depressive symptoms	.14	-.25	-.10	-.21	.21	-.46	-.23	.55										-.06		-.02	-.02			.02	
	**REL MAINTENANCE PROCESSES**																									
9	Δ Learning new things @ P	-.09	.22	.07	.30	-.18	.17	.21	.04	-.05		.09	.06	.08				.01	.01						-.04	
10	Δ Reflecting on your own behv	-.08	.19	.29	.22	-.06	.10	.15	-.03	-.05	.33		.00	.15				.09	.04						-.01	
11	Δ Talking about your rel	-.03	.11	.20	.14	-.07	.14	.23	-.09	-.15	.29	.21			.03		.09	.00	.03		.11	.04			-.03	
12	Δ Quality time together	-.26	.69	.34	.67	-.37	.29	.27	-.13	-.19	.37	.43	.24			.04	.13	.06	.07		.04	.06			-.04	
13	Δ Telling, "I love you"	-.13	.39	.23	.34	-.25	.09	.17	-.02	-.16	.20	.12	.26	.32			.07		.06		.08				-.06	
	**CONNECTIVE REL PROCESSES**																									
14	Δ Emotional support	-.17	.40	.19	.38	-.32	.26	.18	-.17	-.19	.22	.22	.18	.37	.08		.07		.07						-.01	
15	Δ Attentive rel awareness	-.17	.37	.27	.40	-.19	.22	.16	-.13	-.18	.30	.29	.37	.51	.31	.29		.14	.06			.04	-.05		-.18	
16	Δ Gratitude toward partner	-.31	.64	.38	.55	-.47	.21	.13	-.06	-.14	.29	.38	.28	.56	.23	.33	.51		.13				-.12			
17	Δ PP responsiveness	-.30	.55	.30	.47	-.43	.23	.17	-.07	-.27	.25	.31	.28	.51	.31	.40	.40	.50			.02	.05	-.03			-.10
	**SEXUAL FUNCTIONING**																									
18	Δ Freq sexual activity	-.11	.14	.03	.14	-.06	.01	.02	.08	.01	.07	-.02	.12	.16	.09	-.11	.09	.08	.10		.11	.11				
19	Δ Freq physical affection	-.26	.32	.15	.29	-.22	.21	.27	-.16	-.18	.12	.02	.26	.35	.20	.05	.20	.20	.21	.44		.22			-.03	
20	Δ Sexual satisfaction	-.19	.37	.17	.31	-.22	.27	.27	-.12	-.20	.17	.08	.24	.35	.15	.12	.24	.23	.28	.31	.47					-.01
	**DETACHING REL PROCESSES**																									
21	Δ Failing to express gratitude	.03	-.24	-.17	-.41	.25	-.10	-.07	.08	.14	-.23	-.19	-.13	-.30	-.16	-.20	-.35	-.44	-.35	.01	-.12	-.02				
22	Δ Negative conflict behavior	.27	-.19	-.18	-.07	.37	-.15	-.03	.15	.06	.05	-.06	-.03	-.13	-.11	-.25	-.14	-.13	-.14	.00	-.07	-.06	.12		.06	
23	Δ Distraction from rel	.27	-.30	-.20	-.31	.35	-.17	-.19	.20	.25	-.24	-.24	-.29	-.36	-.31	-.28	-.47	-.37	-.28	-.03	-.17	-.19	.27	.35		
24	Δ PP insensitivity	.05	-.45	-.15	-.31	.31	-.27	-.17	.19	.15	-.06	-.13	-.06	-.24	-.17	-.30	-.15	-.32	-.36	.01	-.06	-.21	.05	.11	.22	
	**CHANGE IN WOMEN**																									
	RELATIONSHIP FUNCTIONING																									
0	Baseline rel satisfaction	.58	-.26	-.17	-.13	.24	-.02	-.03	.07	.10	-.15	-.13	-.12	-.22	-.13	-.10	-.12	-.18	-.20	-.11	-.13	-.08	.08	.18	.25	.12
1	Δ Relationship satisfaction	-.20	.36	.26	.27	-.35	.08	.06	-.07	-.12	.15	.13	.17	.27	.18	.34	.07	.24	.26	.06	.08	.16	-.11	-.22	-.18	-.26
2	Δ Dedication to relationship	-.13	.25	.18	.16	-.17	.09	.00	-.03	-.06	.08	-.05	.02	.09	.17	.21	.13	.12	.28	.03	-.02	.20	.06	.07	.04	-.16
3	Δ Positive rel qualities	-.13	.24	.17	.22	-.12	.04	.03	-.18	-.13	.07	.16	.21	.23	.10	.17	.06	.14	.13	.12	.09	.20	-.06	-.08	-.10	-.10
4	Δ Negative rel qualities	.09	-.27	-.12	-.34	.28	-.02	-.02	.03	-.01	-.13	-.06	-.15	-.20	-.22	-.20	-.09	-.10	-.12	-.07	-.13	-.21	.04	.08	.13	.20
	INDIVIDUAL FUNCTIONING																									
5	Δ Vitality	-.01	.26	.12	.17	-.16	.00	-.10	-.04	-.05	.12	-.06	.18	.15	.15	.15	.15	.17	.11	.19	.23	.28	-.21	-.15	-.04	-.09
6	Δ Quality of life	-.11	.18	.00	.07	-.12	-.01	.10	-.04	.02	-.07	-.08	.08	.02	.18	.01	.04	.10	-.01	.07	.18	.14	-.03	-.03	.12	-.09
7	Δ Psychological distress	.09	-.26	-.12	-.21	.17	.09	-.01	.10	.12	-.08	-.16	-.15	-.22	-.18	-.28	-.10	-.14	-.14	-.04	-.21	-.18	.29	.08	.15	.06
8	Δ Depressive symptoms	.07	-.29	-.15	-.31	.17	.04	-.05	.13	-.04	-.02	-.12	-.06	-.21	-.24	-.06	-.09	-.22	-.11	-.10	-.25	-.09	.21	.15	.02	.09
	REL MAINTENANCE PROCESSES																									
9	Δ Learning new things @ P	-.07	.20	.17	.11	-.07	.04	-.01	.00	-.11	.20	.23	.26	.14	.22	.18	.07	.11	.16	.02	-.03	.18	-.02	.03	-.05	.00
10	Δ Reflecting on your own behv	.01	-.07	-.06	-.06	.04	-.09	.02	-.02	.00	.02	.09	.20	-.02	.11	.00	.03	.10	.00	.06	.02	.13	-.11	.04	.03	.04
11	Δ Talking about your rel	.01	-.06	.05	-.06	.06	-.12	.04	.10	.04	.07	.14	.30	.00	.07	-.03	.08	-.02	-.07	.11	.06	.24	-.08	-.02	.07	.04
12	Δ Quality time together	-.17	.31	.19	.27	-.19	.11	.14	-.14	-.08	.09	.10	.22	.28	.24	.10	.12	.22	.16	.11	.17	.28	-.09	-.06	-.11	-.11
13	Δ Telling, "I love you"	-.08	.23	.18	.24	-.16	.12	.16	-.01	-.03	.19	.12	.17	.22	.23	.17	.20	.17	.13	.13	.08	.21	-.10	-.22	-.19	-.10
	CONNECTIVE REL PROCESSES																									
14	Δ Emotional support	.04	.25	.19	.18	-.12	.06	.10	-.04	-.06	-.02	.08	.09	.12	.14	.21	.10	.16	.17	.03	.05	.16	-.04	.07	.05	-.19
15	Δ Attentive rel awareness	-.15	.20	.04	.11	-.29	.03	.08	-.04	.03	.22	.12	.28	.20	.15	.18	.20	.27	.11	.19	.23	.32	-.09	-.10	-.20	-.05
16	Δ Gratitude toward partner	-.19	.19	.08	.12	-.31	-.02	.05	-.04	-.08	.16	.10	.25	.16	.25	.19	.16	.20	.26	.11	.12	.15	-.16	-.17	-.21	-.06
17	Δ PP responsiveness	-.10	.29	.09	.18	-.26	.07	-.06	-.15	-.09	.12	.10	.12	.18	.05	.18	.18	.18	.21	.00	-.06	.08	-.30	-.18	-.23	-.24
	SEXUAL FUNCTIONING																									
18	Δ Freq sexual activity	-.14	.17	.08	.06	-.02	.06	.12	.04	-.01	.08	-.09	-.03	.09	.10	-.04	-.05	.07	.00	.17	.51	.29	-.11	-.08	.08	-.01
19	Δ Freq physical affection	-.17	.22	.09	.09	-.11	.20	.22	-.04	-.04	.25	.11	.17	.31	.08	.08	.18	.16	.05	.19	.62	.38	-.15	-.13	-.14	-.07
20	Δ Sexual satisfaction	-.25	.19	.09	.13	-.19	-.03	.08	.03	-.02	.18	.06	.23	.19	.02	.05	.04	.21	.14	.11	.22	.35	-.07	-.06	-.11	-.11
	DETACHING REL PROCESSES																									
21	Δ Failing to express gratitude	.14	-.10	-.11	-.17	.16	.03	.02	-.01	.09	-.32	-.22	-.24	-.23	-.15	-.21	-.25	-.27	-.25	.11	.03	-.05	.19	.05	.25	-.06
22	Δ Negative conflict behavior	.11	-.15	-.12	-.15	.19	-.02	.06	.11	.07	-.09	-.12	-.11	-.11	-.23	-.18	-.15	-.13	-.22	-.04	.02	-.09	.05	.15	.17	.20
23	Δ Distraction from rel	.15	.06	-.08	-.01	.10	.16	.09	.03	-.02	-.13	-.15	-.22	.07	-.08	-.06	-.16	-.16	-.10	-.04	-.06	-.15	.16	.08	.17	-.08
24	Δ PP insensitivity	.11	-.39	-.15	-.36	.24	-.17	-.13	.14	.08	-.13	-.17	-.20	-.26	-.35	-.25	-.10	-.19	-.26	-.04	-.11	-.10	.20	.22	.25	.40

GENDER — CHANGE IN WOMEN (columns)

CLASS OF VARIABLES		REL FUNCTION					IND FUNCTION				REL MAINTENANCE					CONNECTIVE				SEX FN			DETACHING			
#	Specific variable	0	1	2	3	4	5	6	7	8	9	10	11	12	13	14	15	16	17	18	19	20	21	22	23	24
	**CHANGE IN MEN**																									
	**RELATIONSHIP FUNCTIONING**																									
0	Baseline rel satisfaction	.38																								
1	Δ Relationship satisfaction		.03	.00					-.01										.00							-.02
2	Δ Dedication to relationship		.03																							
3	Δ Positive rel qualities		.00							-.05				.03												-.01
4	Δ Negative rel qualities		.00			.01											-.02	-.11	-.01					.01		
	**INDIVIDUAL FUNCTIONING**																									
5	Δ Vitality																									
6	Δ Quality of life																									
7	Δ Psychological distress																									
8	Δ Depressive symptoms																									
	**REL MAINTENANCE PROCESSES**																									
9	Δ Learning new things @ P														.00						.04	.01	-.12			
10	Δ Reflecting on your own behv										.01												-.02			
11	Δ Talking about your rel				.03						.03		.07				.01					.04	-.04		-.02	
12	Δ Quality time together		.01		.00									.02							.01					
13	Δ Telling, "I love you"					-.04	.01				.00			.04				.05								-.02
	**CONNECTIVE REL PROCESSES**																									
14	Δ Emotional support		.00																							-.04
15	Δ Attentive rel awareness																						-.05			
16	Δ Gratitude toward partner																.02						-.01			
17	Δ PP responsiveness			.01																		.02	.00			
	**SEXUAL FUNCTIONING**																									
18	Δ Freq sexual activity																			.04						
19	Δ Freq physical affection																.01			.17	.26					
20	Δ Sexual satisfaction						.03							.01			.05				.02	.14				
	**DETACHING REL PROCESSES**																									
21	Δ Failing to express gratitude						-.02		.08										-.01							
22	Δ Negative conflict behavior																							.03		.05
23	Δ Distraction from rel	.01															-.05						.00			.06
24	Δ PP insensitivity		-.04																-.01							.11
	**CHANGE IN WOMEN**																									
	RELATIONSHIP FUNCTIONING																									
0	Baseline rel satisfaction		-.18			.02																-.01				
1	Δ Relationship satisfaction	-.49		.03	.24	-.16			-.03		.01			.10		.09		.06	.17			.16		-.02		-.02
2	Δ Dedication to relationship	-.20	.42		.06	-.03																				
3	Δ Positive rel qualities	-.30	.69	.39		-.14	.01	.04	-.05	-.02	.10			.11	.04	.06	.00		.16		.03				-.07	
4	Δ Negative rel qualities	.26	-.57	-.36	-.52		-.01		.10	.02				-.09	-.03			-.07	-.08		-.01		.02	.06		.17
	INDIVIDUAL FUNCTIONING																									
5	Δ Vitality	-.06	.28	.14	.32	-.32		.13	-.20	-.19						.05	.01	.02				.10				
6	Δ Quality of life	-.08	.16	.18	.32	-.18	.42			.00																
7	Δ Psychological distress	.30	-.39	-.15	-.36	.42	-.59	-.26		.27							-.02				-.03		.05			
8	Δ Depressive symptoms	.11	-.19	-.16	-.30	.34	-.54	-.33	.63					-.01			-.01									
	REL MAINTENANCE PROCESSES																									
9	Δ Learning new things @ P	-.15	.38	.27	.43	-.34	.17	.22	-.21	-.11		.05	.16	.15				.02	.03					-.04		
10	Δ Reflecting on your own behv	.02	.18	.04	.21	-.18	.12	.20	-.08	-.05	.33		.17	.07		.02		.04								
11	Δ Talking about your rel	.01	.19	.10	.32	-.23	.26	.15	-.13	-.21	.44	.46					.09	.02			.00	.06			-.09	
12	Δ Quality time together	-.31	.58	.28	.57	-.52	.26	.30	-.29	-.27	.49	.39	.39		.02	.00		.12	.08		.05	.11				
13	Δ Telling, "I love you"	-.09	.30	.19	.30	-.33	.03	.12	-.04	-.07	.20	.09	.21	.29												
	CONNECTIVE REL PROCESSES																									
14	Δ Emotional support	-.09	.40	.30	.40	-.30	.31	.30	-.23	-.13	.27	.24	.16	.35	.17			.05								
15	Δ Attentive rel awareness	-.14	.39	.26	.38	-.36	.33	.28	-.28	-.27	.28	.33	.45	.39	.11	.24		.20	.02		.03	.02	-.03		-.32	
16	Δ Gratitude toward partner	-.25	.53	.36	.46	-.49	.32	.23	-.27	-.18	.38	.36	.41	.57	.23	.38	.58		.09		.04	.06	-.17	-.08	-.01	
17	Δ PP responsiveness	-.31	.60	.22	.56	-.48	.26	.15	-.24	-.17	.37	.24	.26	.50	.23	.32	.36	.47						.00		-.18
	SEXUAL FUNCTIONING																									
18	Δ Freq sexual activity	-.15	.20	.05	.25	-.02	.21	.21	-.11	-.11	.06	-.03	.14	.22	.19	.09	.11	.12	.08		.16	.17				
19	Δ Freq physical affection	-.23	.26	.01	.30	-.22	.23	.14	-.24	-.19	.08	.05	.24	.30	.19	.04	.26	.26	.15	.55		.08				
20	Δ Sexual satisfaction	-.28	.53	.19	.42	-.41	.37	.25	-.29	-.12	.34	.32	.41	.54	.19	.31	.44	.47	.40	.40	.38				-.13	
	DETACHING REL PROCESSES																									
21	Δ Failing to express gratitude	.11	-.26	-.12	-.23	.30	-.27	-.08	.31	.24	-.26	-.17	-.21	-.28	-.13	-.15	-.36	-.39	-.21	.09	-.17	-.20		.00	.14	
22	Δ Negative conflict behavior	.14	-.35	-.27	-.31	.45	-.22	-.24	.18	.15	-.28	-.27	-.20	-.32	-.21	-.29	-.29	-.44	-.31	.07	-.04	-.15	.19			.01
23	Δ Distraction from rel	.17	-.34	-.20	-.37	.33	-.27	-.06	.24	.19	-.26	-.31	-.42	-.36	-.10	-.24	-.61	-.45	-.28	-.06	-.15	-.41	.46	.26		
24	Δ PP insensitivity	.30	-.44	-.22	-.36	.57	-.13	-.16	.19	.12	-.27	-.15	-.15	-.31	-.25	-.23	-.21	-.32	-.50	.00	-.18	-.26	.11	.31	.05	

Supplemental Online Figure S7. *Bivariate Correlations (below diagonal) and Edge Weights (above diagonal) among HLM Linear Slopes Estimated Dyadically. NOTE: Edge weights represent unique associations between each pair of variables (nodes). Specifically, they are partial correlations between each pair of variables controlling for all other variables in the model.*

CORRELATIONS (below diagonal) AND EDGE-WEIGHTS (above diagonal) AMONG HLM LINEAR SLOPES OVER TIME

GENDER	SLOPES IN MEN																SLOPES IN WOMEN															
CLASS OF VARIABLES	REL FN		IND FN		REL MTN		CONNECTIVE				SEX FN			DETACHING			REL FN		IND FN		REL MTN		CONNECTIVE				SEX FN			DETACHING		
# Specific variable	1	2	3	4	5	6	7	8	9	10	11	12	13	14	15	16	17	18	19	20	21	22	23	24	25	26	27	28	29	30	31	32
**SLOPES IN MEN**																																
RELATIONSHIP FUNCTIONING																																
1 INTERCEPT: Rel satisfaction				.00		.04			-.05			.04		.04	.11	.10	.65						.03						.00			
2 SLOPE: Rel satisfaction	-.19		.13			.34	.06	.11	.22	.21		.01	.09	-.02		-.08		.03				.04										
INDIVIDUAL FUNCTIONING																																
3 SLOPE: Vitality	-.10	.44		-.31		.07		.08					.04		-.02													.05				
4 SLOPE: Psychological distress	.16	-.24	-.46												.07	.14																
REL MAINTENANCE PROCESSES																																
5 SLOPE: Learning new things @ P	-.03	.27	.20	-.03		.11	.04	.06	.12	.01	.06													.00								
6 SLOPE: Quality time together	.04	.70	.39	-.12	.36		.09	.13		.12		.25	.03		-.06					-.01												
CONNECTIVE REL PROCESSES																																
7 SLOPE: Emotional support	-.02	.44	.23	-.11	.26	.42		.04	.10	.16					-.03			.02									-.04					
8 SLOPE: Attentive rel awareness	-.16	.60	.38	-.16	.34	.56	.39		.23	.09			.06		-.22			.01				.03	.03									
9 SLOPE: Gratitude toward partner	-.26	.61	.28	-.10	.36	.48	.41	.61		.04		.03			-.15	-.02					.01	.02		.01				.01				
10 SLOPE: PP responsiveness	-.08	.62	.30	-.17	.28	.55	.45	.50	.47			.01	.10	-.01		-.19			.00													
SEXUAL FUNCTIONING																																
11 SLOPE: Freq sexual activity	.03	.04	-.03	.00	.18	.17	.01	.05	.02	.08		.15	.03																			
12 SLOPE: Freq physical affection	.11	.35	.17	-.05	.18	.46	.20	.27	.28	.29	.27		.06																			
13 SLOPE: Sexual satisfaction	-.10	.40	.26	-.07	.16	.36	.11	.35	.28	.37	.16	.26											.03					.03	.15			
DETACHING REL PROCESSES																																
14 SLOPE: Negative conflict behavior	.17	-.20	-.12	.10	-.03	-.13	-.08	-.16	-.12	-.18	-.06	-.02	-.12		.11	.03		-.02		.01								-.01		-.37		.00
15 SLOPE: Distraction from rel	.31	-.41	-.28	.25	-.23	-.39	-.30	-.52	-.48	-.31	.04	-.13	-.19	.26		.02		-.02														
16 SLOPE: PP insensitivity	.28	-.38	-.16	.30	-.07	-.14	-.23	-.22	-.31	-.42	.00	-.05	-.18	.18	.27											-.07						
**SLOPES IN WOMEN**																																
RELATIONSHIP FUNCTIONING																																
17 INTERCEPT: Rel satisfaction	.79	-.04	-.04	.11	-.01	.01	.01	-.08	-.10	-.05	-.01	.00	-.03	.12	.20	.14				.03		.03			-.05			.02		.08	.04	.21
18 SLOPE: Rel satisfaction	-.17	.29	.13	-.12	.04	.20	.20	.28	.24	.21	-.04	.06	.21	-.15	-.23	-.20	-.25		.08	-.04	.02	.20	.04		.17	.24			.15		-.11	-.11
INDIVIDUAL FUNCTIONING																																
19 SLOPE: Vitality	-.08	.19	.02	-.07	.06	.17	.08	.17	.13	.17	.06	.08	.17	-.09	-.11	-.09	-.13	.43		-.37		.03	.08	.12	.03			.04	.03		-.03	
20 SLOPE: Psychological distress	.14	-.14	-.01	.01	-.01	-.16	-.10	-.08	-.07	-.14	-.03	-.02	-.08	.12	.10	.03	.25	-.36	-.55		-.01				-.01			-.04		.01		.17
REL MAINTENANCE PROCESSES																																
21 SLOPE: Learning new things @ P	-.03	.19	.08	-.08	.12	.09	.12	.15	.19	.14	-.04	-.03	.06	.00	-.08	-.11	.01	.37	.18	-.21		.17	.05		.06	.11	.04		.02			
22 SLOPE: Quality time together	-.06	.30	.17	-.11	.08	.23	.11	.28	.27	.20	-.06	.04	.21	-.03	-.19	-.20	.00	.61	.36	-.23	.44		.04	.10	.09	.13		.20	.10			
CONNECTIVE REL PROCESSES																																
23 SLOPE: Emotional support	.07	.14	.08	-.01	.08	.13	.11	.21	.12	.14	-.06	.06	.21	-.03	-.07	-.07	.02	.39	.31	-.17	.28	.36		.05	.09	.09			.05			
24 SLOPE: Attentive rel awareness	-.17	.17	.13	-.06	.14	.13	.13	.18	.21	.14	.01	.05	.20	-.08	-.15	-.15	-.16	.45	.41	-.27	.28	.48	.34		.21	.04			.11		-.29	
25 SLOPE: Gratitude toward partner	-.22	.17	.13	-.08	.12	.12	.11	.21	.18	.14	-.07	.01	.11	-.03	-.20	-.15	-.28	.62	.39	-.32	.37	.54	.40	.59		.19			.01		-.18	-.01
26 SLOPE: PP responsiveness	-.13	.24	.13	-.14	.10	.16	.18	.20	.20	.17	-.02	.01	.11	-.06	-.17	-.26	-.15	.64	.35	-.28	.40	.55	.39	.46	.60							-.20
SEXUAL FUNCTIONING																																
27 SLOPE: Freq sexual activity	.04	.09	.07	.04	.02	.02	-.11	.07	.09	.02	-.05	.03	.17	-.10	-.02	-.07	.07	.10	.12	-.14	.21	.24	.11	.13	.08	.10		.32	.13		.02	
28 SLOPE: Freq physical affection	-.07	.21	.20	-.13	.08	.14	.02	.17	.19	.06	.00	.00	.23	-.13	-.12	-.11	.07	.30	.27	-.23	.22	.45	.17	.27	.20	.22	.48		.12			
29 SLOPE: Sexual satisfaction	-.17	.18	.12	-.03	.06	.11	.00	.19	.21	.10	.01	.06	.34	-.07	-.14	-.09	-.15	.51	.33	-.24	.29	.48	.32	.44	.43	.37	.34	.41			-.08	
DETACHING REL PROCESSES																																
30 SLOPE: Negative conflict behavior	.12	.12	.09	.05	-.02	.10	.00	.04	.00	.01	.07	.04	.09	-.47	-.06	.07	.20	.06	-.03	.12	-.04	.03	.03	.00	-.08	.00	.03	.10	.07			.01
31 SLOPE: Distraction from rel	.19	-.11	-.08	.06	-.07	-.12	-.11	-.18	-.19	-.13	.01	-.09	-.15	-.05	.13	.05	.24	-.49	-.34	.21	-.26	-.40	-.25	-.59	-.55	-.36	.02	-.14	-.40	.10		
32 SLOPE: PP insensitivity	.28	-.14	-.07	.07	-.06	-.09	-.11	-.10	-.13	-.12	.04	-.07	-.05	.12	.15	.06	.44	-.44	-.24	.38	-.21	-.26	-.23	-.24	-.37	-.48	-.02	-.11	-.26	.14	.22	
